# Anion Exchange Membranes Based on Chemical Modification of Recycled PET Bottles

**DOI:** 10.1021/acsapm.3c01391

**Published:** 2023-08-30

**Authors:** Varun Donnakatte Neelalochana, Eleonora Tomasino, Rosa Di Maggio, Oscar Cotini, Paolo Scardi, Stefano Mammi, Narges Ataollahi

**Affiliations:** † Department of Civil, Environmental, and Mechanical Engineering, University of Trento, 38123 Trento, Italy; ‡ Department of Chemical Sciences, University of Padova, Via Marzolo 1, 35131 Padova, Italy

**Keywords:** PET bottle, anion exchange membrane, chemical modification, electrochemical application, cationic functional group, amination syntheses, alkaline stability, oxidation stability, sustainability, circular economy

## Abstract

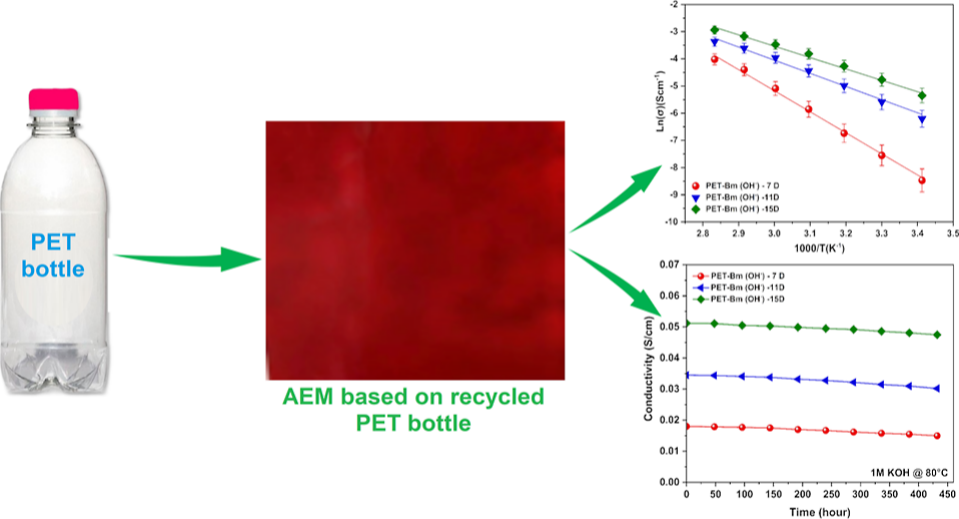

This study presents an innovative and effective solution for recycling PET bottles as raw for producing anion exchange membranes (AEMs) for electrochemical applications. This approach reduces the demand for pristine materials, a key principle of the circular economy and sustainability. PET was subjected to chemical modification by introducing cationic functional groups followed by methylation and OH^–^ exchange process. The amination synthesis was optimized based on reaction time. The results indicate that ion exchange capacity, water uptake, and swelling ratio properties mainly depend on the degree of cationic functionalization. The optimized AEM exhibits ionic conductivity of 5.3 × 10^–2^ S·cm^–1^ and alkaline stability of 432 h in 1 M KOH at 80 °C. The membrane properties before and after the alkaline treatment were investigated using Fourier-transform infrared spectroscopy, thermogravimetric analysis, and scanning electron microscopy analysis. Computational chemistry analysis was employed to gain further insights into the membrane degradation mechanisms and pathways under alkaline conditions. This research and its findings are a step toward using recycled materials in the field of AEM technology.

## 1 Introduction

The membrane is an essential part of a wide range of electrochemical devices, such as fuel cells, flow batteries, electrolyzers, and water desalination through electrodialysis.^1−3^ Although renewable energy has been vigorously developed, the reliable operation of the power system faces significant challenges due to its unique interruption and instability. This situation leads to temporal and spatial gaps between the availability and consumption of energy.^4^ Hence, electrochemical devices based on membranes can serve as a reliable approach to attaining efficient and stable renewable energy. Until now, various polymers have been used as anion exchange membranes (AEMs).^5^ Polymers (typically nonfluorine-based) act as an electrolyte and play a crucial role in transferring hydroxide ions (OH^–^) in AEM fuel cells or AEM water electrolyzers.^6,7^ However, many technological aspects of the production of AEMs are still obscure and far from being achieved, particularly those related to durability and OH^–^ conductivity.^8^

This research focuses on synthesizing an anion exchange membrane made of modified PET from recycled bottles. PET is a semicrystalline thermoplastic polyester synthesized by polycondensation of ethylene glycol and dimethyl terephthalate,^9^ which makes it thermally stable up to 400 °C and resistant to hydrolytic degradation.^10^ It also shows excellent optical properties, high transparency, and good mechanical properties, together with gas-barrier ability, low density, and moderate chemical resistance.^11^ However, PET has potential drawbacks arising from low surface free energy and high chemical inertness, resulting in poor wettability, printability, and stickiness.^12^ PET has been used as an intermediate material for tissue scaffolds, filtration devices, and vascular membrane prostheses due to its distinct structure and physicochemical characteristics.^13^ It is broadly used in textiles, fibers, food packaging, and soft drink bottles.^14^ The waste of PET bottles causes serious environmental issues, as bottles will require many years to degrade. According to the literature,^15−17^ the global production of plastics is increasing quickly and is predicted to reach over 34 billion metric tons in 2050. Europe ranked fourth in the world for plastic materials, with 55 million tons produced. However, 29 million tons of post-consumer plastic bottles were collected from the packaging industry, with 34.6% being sent to recycling facilities, 42.2% to energy recovery activities, and 23.4% to landfills.^17^ Recycling PET bottles is a suitable approach to minimize their environmental footprint.^18^ The chemical modification of PET bottles is a possible strategy that tries to alter the structure of polymers by introducing new functional groups, which impart new characteristics and properties.^19^

The modification of commercial PET as the proton exchange membrane (PEM) using the ultraviolet (UV) radiation grafting technique has been the subject of several studies.^20−22^ Among them, Ahmed et al.^20^ showed that grafting allyl acetate onto PET films results in considerable improvement over the nonfluorinated membrane for fuel cells. Abdel-Hady et al.^21^ grafted styrene onto the PET film with different grafting degrees. The fabricated membrane exhibited a conductivity of 5.8 × 10^–3^ S·cm^–1^ and effective methanol permeability, demonstrating the feasibility of this membrane in direct methanol fuel cells. ElHakim et al.^22^ achieved a proton conductivity of 6.03 × 10^–3^ S·cm^–1^ through graft copolymerization of glycidyl methacrylate onto PET film, followed by sulfonation. Although the modified PET membrane’s conductivity was lower compared to the Nafion membrane, the results were encouraging, suggesting the potential of the modified PET membrane in fuel cells.

In this study, the chemical modification was carried out by reacting a PET waste bottle with ethylene glycol, followed by introducing a cationic functional group (−NH_2_). The strong point of this study is the conversion of modified PET to anion exchange membranes by the incorporation of the quaternary ammonium (QA) group via the methylation and OH^–^ exchange process considering the novelty of this study compared to the state-of-the-art described above. Beyond its evident focus on the recycling issue, the utilization of PET as the raw material derived from discarded bottles offers substantial opportunities in terms of the inherent chemical structure of the polymer, which serves as a favorable platform for the subsequent grafting of chemical functionalities essential for ion exchange. Adopting a prestructured PET polymer can save time, energy, and cost in the synthesis and membrane preparation compared with commercial PET, which costs around US$0.60/g.^23^ Furthermore, it can be a suitable replacement for the costly AEM-FAS-30 ($21/cm^2^)^24^ and Nafion ($25/cm^2^)^25^ membranes.

In this study, the validity of the predicted chemical structure of the compound was proved through NMR, Fourier transform infrared spectroscopy (FTIR), and quantum chemical calculations. The potential of modified PET membranes in electrochemical applications was evaluated by measuring OH^–^ conduction. Additionally, the effect of the amination period on the ion exchange capacity, water uptake, swelling ratio, and chemical durability was determined. Moreover, insightful computational chemistry analysis was employed to understand membrane degradation under alkaline conditions. The results demonstrated the practicality and viability of using recycled PET bottles as AEM.

## 2 Experimental Section

### 2.1 Reagents

Ethylene glycol (EG, 99.8%), 1,2-diaminopropane (DAP, 99%), zinc acetate (ZA, 99.9%), 1,1,3,3,3-hexafluoroisopropanol (HFIP, 99%), iodomethane (IM, 99%), potassium hydroxide (KOH, 90%), potassium chloride (KCl, 99%), silver nitrate (AgNO_3_, 99%), sodium nitrate (NaNO_3_, 99%), ferrous sulfate (FeSO_4_, 99.8%), potassium chromate (K_2_CrO_4_, 99%) and hydrogen peroxide (H_2_O_2,_ 99.8%) were acquired from Sigma-Aldrich. Hydrochloric acid (HCl, 99.9%), ethanol (99.8%), sodium hydroxide (NaOH, 98.8%), and acetone (99.5%) were purchased from Honey Well. Polyketone (PK) was received from Hyosung Co. Ltd. (Seoul, South Korea). Ultra-pure water (H_2_O—LF < 1 μS·cm^–1^–0.4 μm) is collected from the Chem Lab NV. PET was recovered from wastewater bottles.

### 2.2 Methods

#### 2.2.1 Chemical Modification of PET

**Figure 1:**
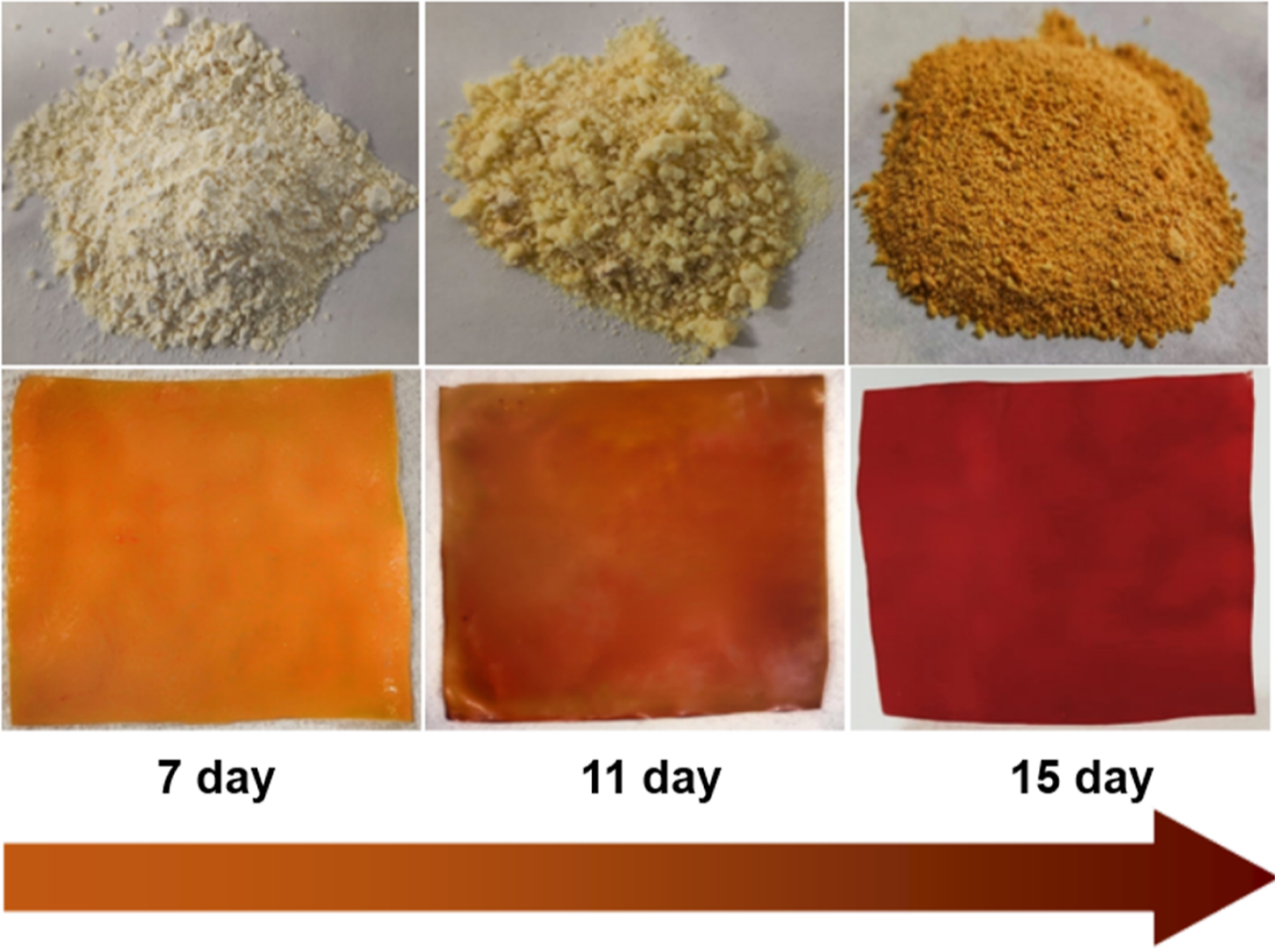
Picture of the PET-B products and membranes (7, 11, and 15 days; thickness: 20 μm).

**Scheme 1:**
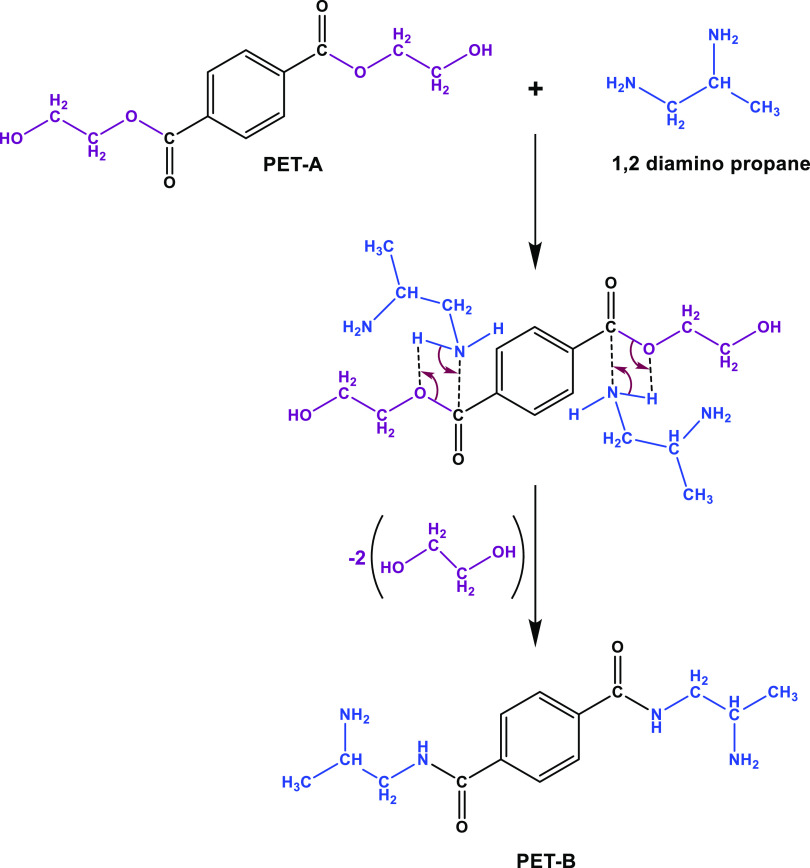
Reaction Mechanism of 1,2 Diaminopropane with PET-A Forming PET-B (step-2)

Chemical modification of PET bottles was carried out in two steps. The first synthesis step was performed according to Mendiburu-Valor et al.^26^ PET bottles were cut into small flakes, washed with ultra-pure water, and dried for 3 h at 40 °C. The PET flakes were then mixed with ethylene glycol as a degrading agent (ratio of PET/EG 1:3 w/w) and zinc acetate as the catalyst (0.2% of PET weight) inside a two-necked flask connected to a condenser. This synthesis was carried out from 160 to 190 °C for 1–5 h to find the best conditions to maximize the yield. Finally, the obtained product was rinsed with ultra-pure water and ethanol, filtered, and dried for 8 h at 60 °C. A solid white powder was obtained and named PET-A. The detailed mechanism for the formation of PET-A, along with the optimization of the reaction, is shown in the Supporting Information (Scheme S1 and Figure S1). In the second step, PET-A was reacted with 1,2-diaminopropane without any catalyst or solvent under mild conditions. About 5 g of PET-A (obtained at 190 °C for 4 h) was mixed with 15 mL of DAP and stirred at 40 °C at varying intervals for 7–15 days. As the amination reaction proceeded, the solution color changed from light yellow to dark yellow and turned brown at higher reaction times, as shown in Figure 1. In the end, the product (PET-B) was collected, washed with acetone, and then filtered and dried overnight at 60 °C. The reaction mechanism is shown in Scheme 1 and will be discussed in Section 3.1.

#### 2.2.2 Membrane Preparation

**Scheme 2:**
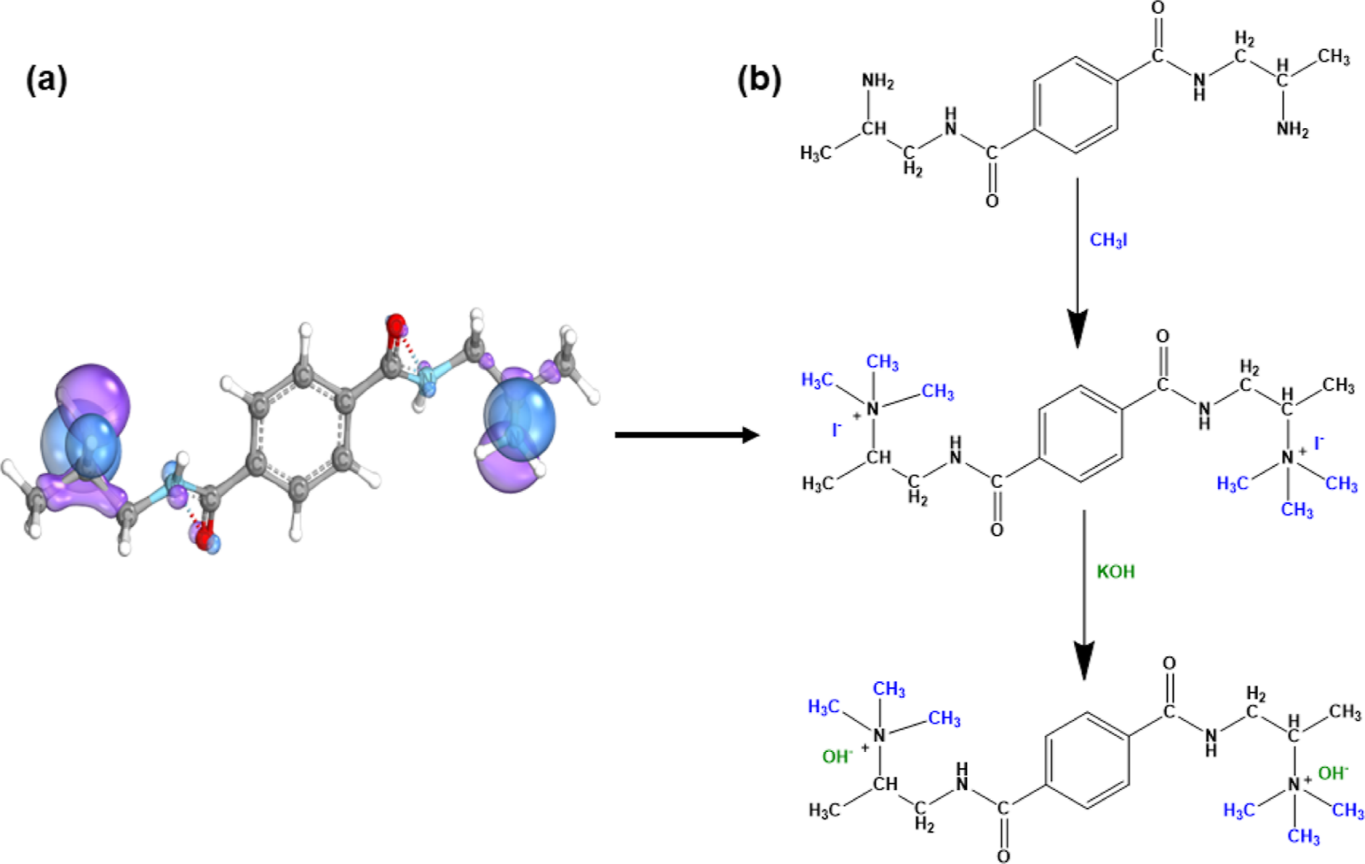
(a) HOMO Orbitals in PET-B Obtained from DFT and (b) Methylation Reaction and OH^–^ Exchange

To prepare the membrane, 0.3 g of PET-B was dissolved in 2 mL of HFIP. Polyketone (20% of PET-B weight) was added as a binder and compatibilizer. This helps to increase the interfacial bonding between the component of the AEM and to improve the compatibility between the hydrophobic part of the polymer matrix and a hydrophilic solvent.^27^ Then, the solution was subjected to 30 min of homogenization using an ultrasonic bath and stirred at 40 °C for 24 h. The obtained mixture was cast into the glass mold and dried for 5 h under vacuum at ambient temperature. Finally, flexible membranes (PET-Bm) were obtained, as shown in Figure 1. The PET-Bm membranes were subjected to a methylation process by immersing in iodomethane at room temperature for 24 h. The methylated membranes (PET-Bm(I)) were washed thoroughly with ultra-pure water to remove excess iodomethane. The OH^–^ exchange was conducted by immersing the PET-Bm(I) in a 1 M KOH solution under a nitrogen atmosphere. The obtained membrane (PET-Bm(OH^–^)) confirms the ease of preparation of AEM from recycled PET bottles. The reaction mechanism is shown in Scheme 2b and will be discussed in Section 3.2.

### 2.3 Characterization and Measurements

A LECO-CHN628 instrument (Saint Joseph, MI, USA) was used for the elemental analysis to determine the amount of nitrogen per gram in the modified PET. NMR spectra were acquired in CDCl_3_ (PET-A) and DMSO-*d*_6_ (PET-B) at 298 K using Bruker 400 Advance III HD spectrometer. The instrument was equipped with a 5 mm multinuclear inverse z-field gradient probe head, operating ^1^H frequency 400.13 MHz and ^13^C frequency 100.62 MHz. The data processing was performed with the Topspin 3.5 software. The ^1^H 1D spectrum was obtained with 16 transients, 32 k data points, 10 ppm spectral width, and 10 s recovery delay, and processed with zero filling, exponential multiplication, and 0.3 Hz line broadening. FTIR was conducted using a PerkinElmer Spectrometer in the wavenumber range between 4000 and 600 cm^–1^: 4 scans with a resolution of 4 cm^–1^. Scanning electron microscopy (SEM) analysis was performed using a JEOL JSM-IT300 LV instrument, complemented by microanalysis using an energy-dispersive X-ray spectroscopy (EDXS) system, specifically the BRUKER QUANTAX with XFlash 630 M, seamlessly integrated with the microscope. The membrane samples were rapidly quenched in liquid nitrogen, manually fractured, and then coated with a thin layer of gold for examination. The thermal stability of samples was evaluated by thermogravimetric analysis (TGA) using the high-resolution modulated Labsys SETARAM thermobalance (Caluire, France). The measurements were conducted using a temperature range of 25 to 600 °C with a 10 °C/min heating rate in the N_2_ atmosphere. The investigation of tensile strength was conducted using an EXSTAR TMA/SS6000 thermo-mechanical analyzer (Seiko Instruments Inc., Chiba, Japan). The samples were subjected to a gradual application of a 6 N tension force until the fracture point was reached. Throughout the testing process, the humidity inside the test chamber was meticulously monitored using a digital hygrometer and actively regulated using a humidifier, ensuring a consistently maintained minimum relative humidity of 90%.

Quantum chemical calculations were carried out with ORCA software^28^ to perform structural analysis of the synthesized PET-B material. To confirm the predicted structure, FTIR, ^1^H NMR, and ^13^C NMR spectra were evaluated via Density Functional Theory (DFT), using Becke-3-Parameter–Lee-Yang-Parr (B3LYP) functional and 6-311G++(d,p) basis set. The charge-dependent Atom-pairwise Dispersion Correction (D4) by Grimme^29^ was used to include the contribution of dispersion forces. Frontier Orbital analysis was performed to individuate the preferential sites for methylation reaction and confirm the methylated structure of PET-B.

The Mohr technique was utilized to determine membrane ion exchange capacity (IEC). The procedure consisted of drying the membranes and immersing them in a 1 mol·L^–1^ KCl for 24 h to ensure that Cl^–^ ions were fully attached to the membrane. The unreacted KCl was removed by washing the membranes with ultra-pure water. The membrane was then placed in a 0.5 mol·L^–1^ NaNO_3_ solution to reach equilibrium and then removed. The titration was conducted with 0.1 mol·L^–1^ AgNO_3_ and K_2_CrO_4_ as an indicator. Finally, a brick-red precipitate of Ag_2_CrO_4_ was formed, indicating the endpoint. The IEC was calculated using Formula 1.
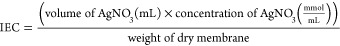
1

To measure the water uptake (WU) and the swelling ratio (SR), the PET-Bm(OH^–^) (2 cm^2^ in size) was dried for 24 h at 40 °C, and the weight and area/thickness of the dry membrane were assessed. Next, the membrane was kept in ultra-pure water at 20, 30, 40, 50, 60, 70, and 80 °C for 24 h. The surface moisture of the membrane was wiped with tissue paper. The weight and area of the wet membranes were then immediately measured. The WU and SR were calculated using formulas 2 and 3.
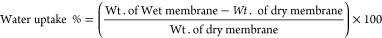
2
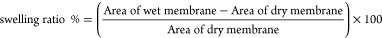
3

The hydration value (λ) measures the number of H_2_O molecules surrounding each QA cation and was determined with the WU and IEC values shown in Formula 4. The molar mass of water is represented as M_H2O_ (18 g/mol).
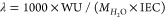
4

The PET-Bm(OH^–^) was hydrated in ultra-pure water for 24 h before conductivity measurement. Then, they were wiped with tissue paper, sandwiched between two circular gold electrodes having a diameter of 1.27 cm, and placed inside a CESH sample holder. The process was performed in a controlled atmosphere glovebox. The sample holder was placed inside Biologic’s intermediate temperature system (ITS) for conductivity measurements in temperature and connected to the SP-150 Biologic (Seyssinet-Pariset, France) instrument. The resistance was measured at temperatures ranging from 30 to 80 °C, with applied frequencies ranging from 1 MHz to 100 Hz and a voltage of 0.01 V. The conductivity was determined using the following Formula 5.
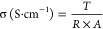
5where *T* is the thickness of the membrane (cm), *R* is the measured resistance (Ω), and *A* is the area (cm^2^). The conductivity activation energy (*E*_a_) was determined using the linear Arrhenius equation *E*_a_ = −*b* × *R*, where *R* is the gas constant (8.13 J/(mol·K)) and *b* can be obtained from the slope of the linear regression of lnσ versus 1000/T.

The alkaline stability of membranes was carried out by immersing PET-Bm (OH^–^) in 1 M KOH at 80 °C for 432 h. In addition, Fenton’s reagent (3 wt % H_2_O_2_ and 4 ppm FeSO_4_) was used to measure the oxidative stability of AEMs, wherein the process was conducted at 80 °C for 120 h. The degraded membrane was characterized using hydroxide conductivity, FTIR, TGA, and SEM analysis. Finally, transition state analysis (TSA) was performed at the B3LYP/6-311G level of theory to investigate different possible degradation mechanisms of the membrane under an alkaline environment and in solvated conditions.

The activation (Δ*E*_a_) and reaction (Δ*E*_R_) energies are computed as the difference in electronic energies, as shown in eqs 6 and 7
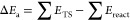
6
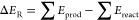
7

## 3 Results and Discussion

### 3.1 Chemical Modification and Elemental Analyses

The depolymerization of PET with ethylene glycol is the starting point of the process, inspired by reference,^26^ and the optimization of the reaction is presented in Figure S1 (step-1) and Table S1. The transformation of PET-A into PET-B involves the introduction of amide and amine groups; only the latter is supposed to be involved in subsequent methylation. According to Scheme 1, this reaction occurs using 1,2-diaminopropane, a key step in increasing the transformation yields with respect to previously used reagents. The mechanism involves the formation of an intermediate amide linkage through a nucleophilic attack of the primary amine groups on ester bonds in PET-A.^30^ The reaction is followed by deprotonation of the hydroxyl group resulting in the final PET-B structure. The structure is confirmed with NMR and supported with FTIR and DFT analysis, as discussed in Section 3.2.

The degree of conversion of the O–C=O group of PET-A into an amide functional group as in PET-B was evaluated through the results of elemental analysis in Table S1. The difference observed between the carbon content of commercial PET and recycled PET bottles may be due to the presence of processing additives, which are not precisely known and may represent a limit to the reproducibility of the process and yield. The increase in nitrogen content is a measure of the transformation. The conversion rate was low since the reaction was carried out without any catalyst, which could speed it up in the case of industrial implementation. A minimum of 7 days was required for 50% conversion and 54% yield, which increased to 70 and 80% after 15 days, and the optimization of the reaction (step-2) is presented in Figure S2, indicating that the amination process highly depends on the reaction time. It seems that an equilibrium state was reached when the reaction time exceeded 15 days; no further conversion was observed, which could be due to the basic nature of amine and the steric hindrances.^31^

### 3.2 Chemical Structure Analysis: NMR, FTIR, and DFT

**Figure 2:**
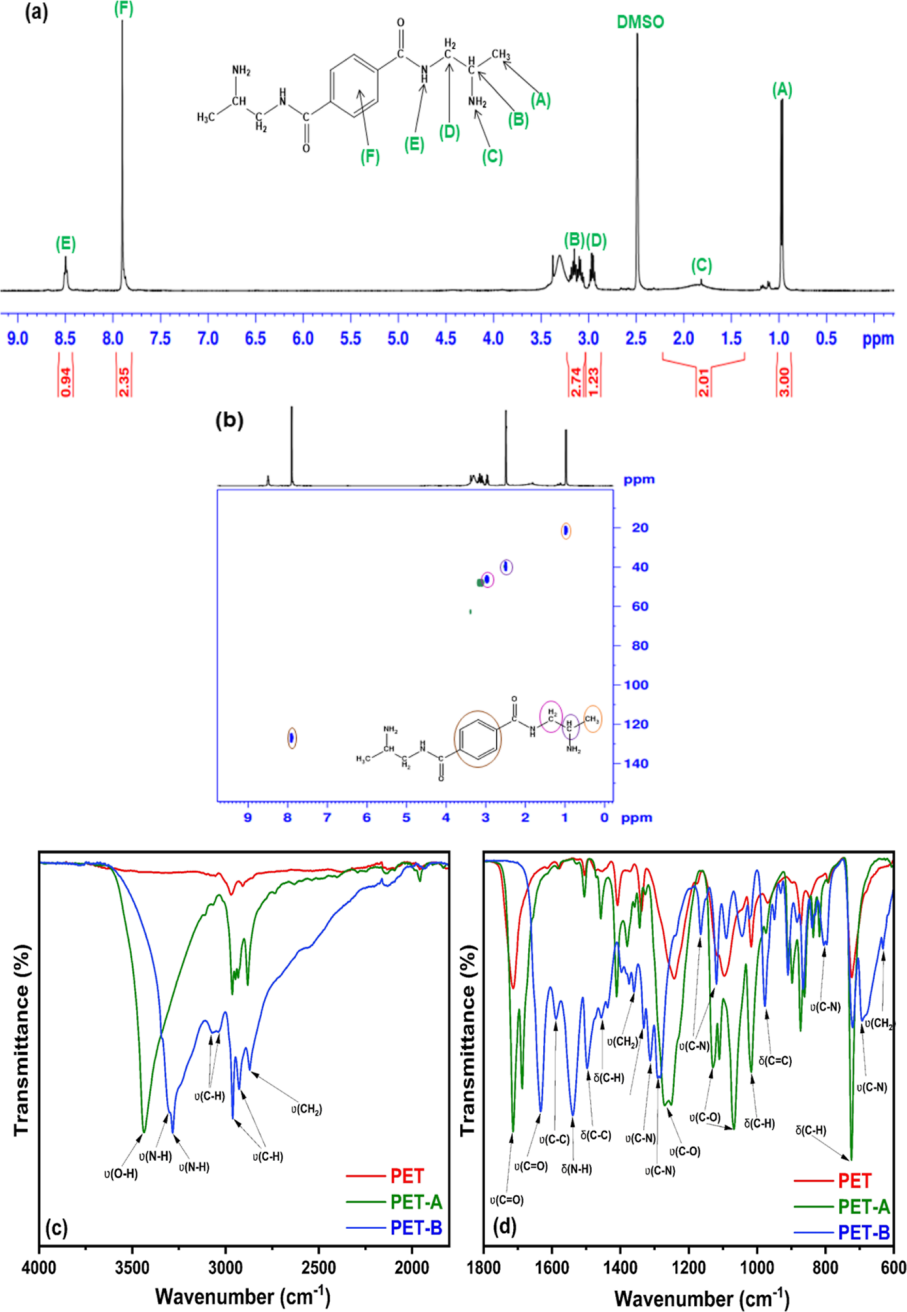
(a) ^1^H NMR and (b) ^13^C NMR spectra for PET-B. FTIR spectrum of PET, PET-A, and PET-B. (c) In the 4000–1900 cm^–1^ range and (d) in the 1800–600 cm^–1^ range.

The ^1^H NMR and ^13^C NMR (Figure S3) spectra of PET-A confirmed the proposed structure (Scheme S1), and the detailed discussion is presented in the Supporting Information. The chemical structure of PET-B was also validated by the ^1^H NMR and ^13^C NMR spectra, as shown in Figure 2a,b. The presence of a sharp doublet at 0.96 ppm corresponds to the protons of the methyl group CH_3_. The broad signal at 1.8 ppm is assigned to the primary amine in the structure. The signal of CH–CH_3_ was observed at 2.95 ppm. The multiple peaks observed at 3.15 ppm are ascribed to the CH_2_–CH group adjacent to the amide bonds. A sharp peak at 7.89 ppm corresponds to the protons of the aromatic rings. The peak of the amide is observed at 8.5 ppm. Its multiplicity (a triplet rather than a doublet) confirms that the amine in position 1 reacted and not in position 2. A sharp peak at 2.49 ppm is also assigned to the DMSO solvent. In the ^13^C NMR spectrum of PET-B (Figure 2b), the signals at 20.8, 40.1, 49.5, and 127.6 ppm correspond to the carbon atoms of the CH_3_, CH, CH_2_ groups and of the aromatic ring, respectively.^32,33^

FTIR analysis provides detailed information about chemical bonds and functional groups in a sample. Figure 2c,d shows the FTIR spectra of three different samples of PET bottle, PET-A, and PET-B. The peaks observed in the spectra are assigned in Table S2. The primary vibration spectra of the PET gave rise to strong bands at 723, 2907, and 2970 cm^–1^, assigned to the C–H bond in the ethyl group.^34^ The C–O stretching vibration of the ester group was observed at 1020, 1093, and 1240 cm^–1^.^34,35^ The C–C stretching vibration of the ring was detected at 1340 and 1408 cm^–1^.^36^ Medium peaks at 1503 and 1588 cm^–1^ were attributed to the C=C aromatic ring.^37^ Lastly, a strong stretching vibration at 1714 cm^–1^ corresponded to the carbonyl group (C=O).^38^ The modification of PET by ethylene glycol revealed the presence of a new functional group, particularly the hydroxyl group observed at 3439 cm^–1^.^39^ The addition of 1,2-diaminopropane to PET-A results in a significant shift from 1714 to 1632 cm^–1^ in the stretching vibration of C=O peak in PET-B.^38,40^ This shift can be attributed to the introduction of the amine group in PET-B, which forms a hydrogen bond with the carbonyl group of PET-A. This interaction decreases the polarity of the carbonyl group, resulting in a downshift of the peak. Additionally, the N–H stretching vibration of the amine group in PET-B was detected at 1539 and 3303 cm^–1^,^37,41^ while peaks at 692, 819, 1165,1283, 1313, and 1540 cm^–1^ correspond to the C–N stretching vibration,^7,42,43^ indicating the successful modification of PET-B. The use of recycled PET bottles with their inherent chemical structure of the polymer proves to be a viable and effective approach for incorporating ion exchange functional groups.

DFT analysis of FTIR and NMR was performed on the single structural unit of PET-B since the structural parameters and electronic properties do not significantly differ from long chains.^44^ Geometry optimization was carried out in vacuum conditions, and the validity of the final structure was assessed by analyzing the vibrational frequencies of the system. A true minimum is reached when all frequencies are positive. FTIR spectrum obtained for the optimized structure is presented in the Supporting Information (Figure S4a). The simulated peaks were consistent with the experimental data, with a slight shift towards lower frequencies. The main peaks, attributed to C=O, N–H, and C–N, were observed at 1627, 1453, and 746 cm^–1^, respectively. The optimized structure of PET-B was subsequently used to calculate ^1^H and ^13^C NMR spectra. ORCA software uses the Gauge-Independent Atomic Orbitals (GIAOs)^45^ method, and Tetramethyl silane (TMS) was applied as a reference to calculate the final chemical shifts. The values, in ppm, are shown in Figure S4b,c and reported in Table S3. The observed peaks in the ^13^C NMR demonstrate a strong agreement with the measured values. The additional peaks observed at 4.34 and 5.32 ppm in ^1^H NMR may be assigned to C–H groups. Some deviations in chemical shifts are expected due to many external factors affecting the experimental measurements, which may also distort or hide peaks.^46^ Overall, the excellent agreement observed between the DFT analysis and the experimental data from FTIR and NMR confirms the accuracy of the predicted structure of PET-B.

The molecular orbital theory is employed to study the reactivity of molecules. The analysis of frontier orbitals (HOMO and LUMO) is a well-established method to individuate the nucleophilic and electrophilic regions, which are crucial sites for the incorporation of quaternary ammonium group through methylation reactions which are subsequently exchanged with hydroxide anions. Specifically, HOMO orbitals identify the nucleophilic regions of the molecule that are more susceptible to electrophilic attack.^47^ The methylation of PET-B occurs via an SN_2_ substitution reaction, where an electrophilic alkylating agent (iodomethane) attacks a nucleophilic group.^48^ By analyzing the frontier orbitals of PET-B, it is possible to predict the structure of the polymer containing QA groups. As depicted in Scheme 2, the distribution of HOMO orbitals around the primary amines (NH_2_) indicates their nucleophilic behavior, confirming the preferential sites for the methylation reaction (Scheme 2b).

### 3.3 TGA Analysis

**Figure 3:**
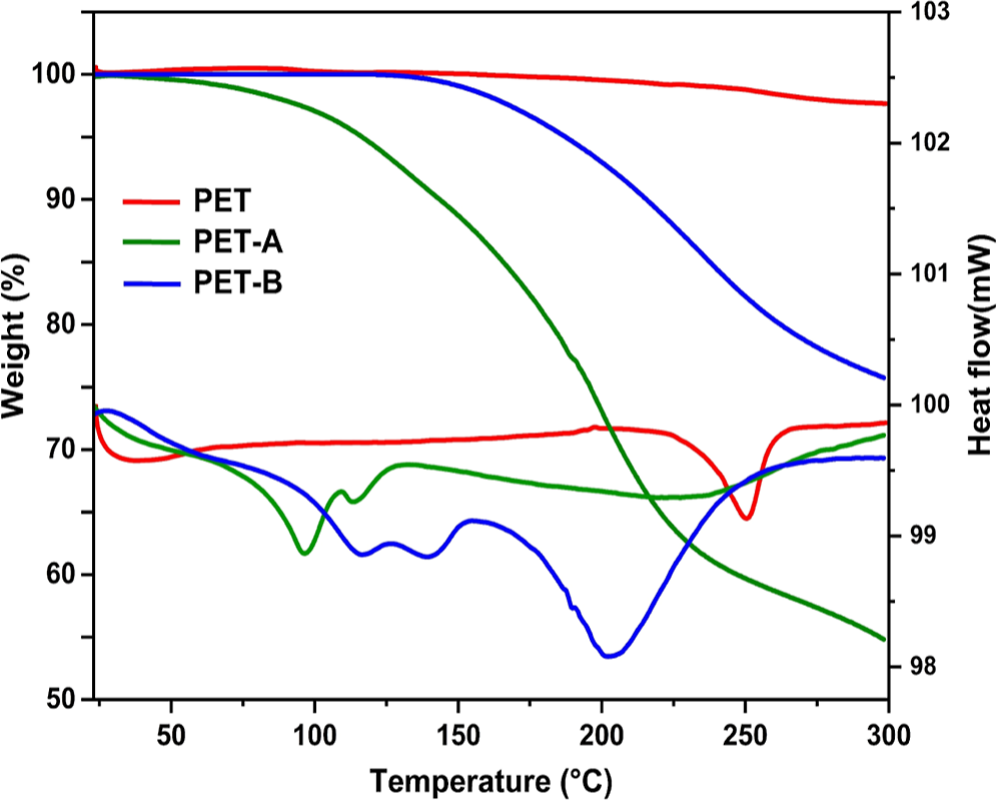
TGA and DTA profiles of PET, PET-A, and PET-B.

The thermal behavior of PET, PET-A, and PET-B was determined by TGA and is reported in Figure 3. PET does not show any signal up to 250 °C, suggesting good thermal stability. At that temperature, a peak of depolymerization and decomposition occurs as shown in the DTA profile, accompanied by 3.3% weight loss in the temperature range of 250–300 °C. The modification of PET induced by ethylene glycol produces PET-A, which already has a glass transition at around 40 °C (as shown in the DTA curve), and around 80 °C begins to degrade up to 50% at 300 °C. In the DTA curve of PET-A, the endothermic peak at 96 °C correspond to the dehydration and evaporation of the low molar weight component of EG, whereas the peak at 115 °C is attributed to the melting point of modified PET.^49,50^ PET-B starts degrading after reaching 150 °C and up to 300 °C. Therefore, as expected, the modifications of the PET result in a worsening of the material’s thermal resistance. At temperatures ranging from 150 to 300 °C, the degradation of the amide functional group, ester group (C–O–C), and unsaturated chain (−C=C−) was observed, leading to a weight reduction of 22% as also indicated in the DTA profile of PET-B. Previous similar works^50^ have proposed a general discussion on the different trends of the curves.

### 3.4 Membrane Characterization

#### 3.4.1 IEC, Water Uptake, Swelling Ratio, and Ionic Conductivity

**Figure 4:**
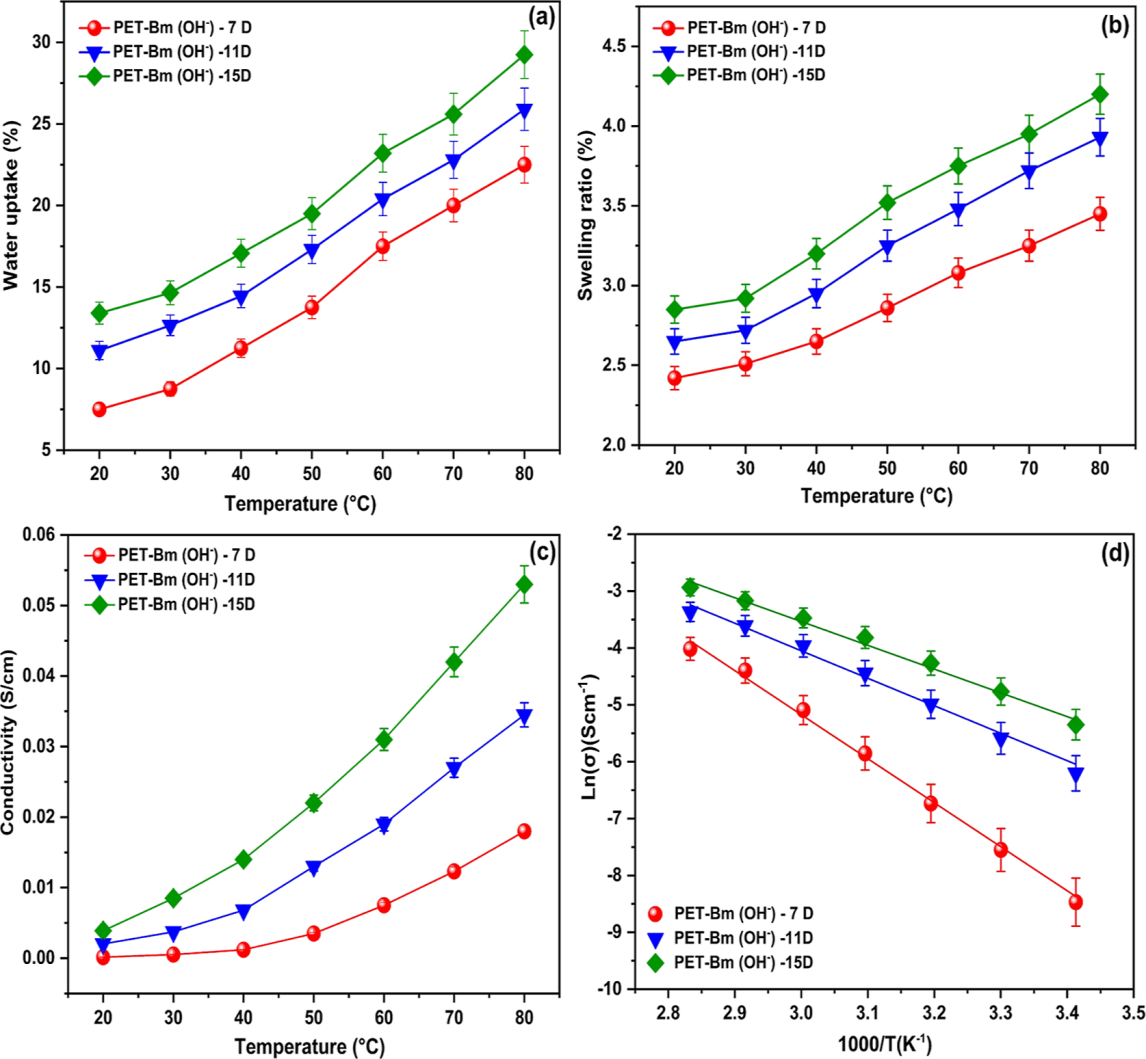
(a) WU and (b) SR of PET-Bm (OH^–^) of amination periods ranging from 7 to 15 days (D) at different temperatures, (c) ionic conductivity for each amination period (7 to 15 D) plotted versus temperature. (d) Arrhenius plot vs temperature.

Our results demonstrate a direct correlation between the content of amine groups with IEC, WU, and SR: the more amine functional groups in the membrane, the higher the value of IEC, WU, and SR (Figure 4a–c, respectively). Their temperature dependence was observed for OH^–^ membrane at different degrees of amination (7, 11, and 15 days). WU values varied from 7.5 to 13.4% and from 22.5 to 29.3% at 20 and 80 °C, respectively (Figure 4a). The values of λ span from 3.4 to 12.04 within the temperature range of 20 to 80 °C, as reported in Table 1, confirming the presence of a higher hydrophilic functional group in the membranes.^51^ All membranes maintained an SR below 5%, which is considered good dimensional stability. These results directly affect the ionic conductivity of membranes. Generally, hydronium ion diffusion and ion conduction are facilitated in highly hydrated membranes.^52^ The conductivity values of the membrane obtained after 7 days or 15 days of amination increased from 1.80 × 10^–2^ S·cm^–1^ to 5.3 × 10^–2^ S·cm^–1^ at 80 °C, respectively (Figure 4c). These findings suggest that the membrane conductivity is influenced by the degree of amination or IEC and also temperature, the latter affecting the mobility of the ions.^53^ The Arrhenius conductivity plots for the PET-Bm(OH^–^) membranes showed a clear linear trend (Figure 4d), a clue for the mechanism of ion hopping conduction.^54^ Furthermore, the lowest activation energy was obtained for membranes with a higher amination period (15D) (Table 1), consistent with the conductivity results. The results obtained fulfill the essential conductivity requirement of the electrochemical application.^55−58^

**Table 1:** Measured Properties of PET-Bm (OH^–^) Based on IEC, WU (%), SR (%) Hydration, and Activation Energy

membrane	IEC (mmol g^–1^)	WU (%)		SR (%)		λ		*E*_a_
PET-Bm(OH^–^)		20 °C	80 °C	20 °C	80 °C	20 °C	80 °C	kJ/mol
7 days (50%)	1.25 ± 0.10	7.50 ± 1.21	22.50 ± 1.30	2.40 ± 0.2	3.45 ± 0.2	3.33	10.00	64.40
11 days (65%)	1.30 ± 0.12	11.11 ± 1.42	25.90 ± 1.25	2.65 ± 0.1	3.90 ± 0.3	4.74	11.07	40.17
15 days (80%)	1.35 ± 0.10	13.40 ± 1.61	29.25 ± 1.12	2.85 ± 0.1	4.20 ± 0.1	5.51	12.04	33.99

#### 3.4.2 Thermal and Mechanical Stability

**Figure 5:**
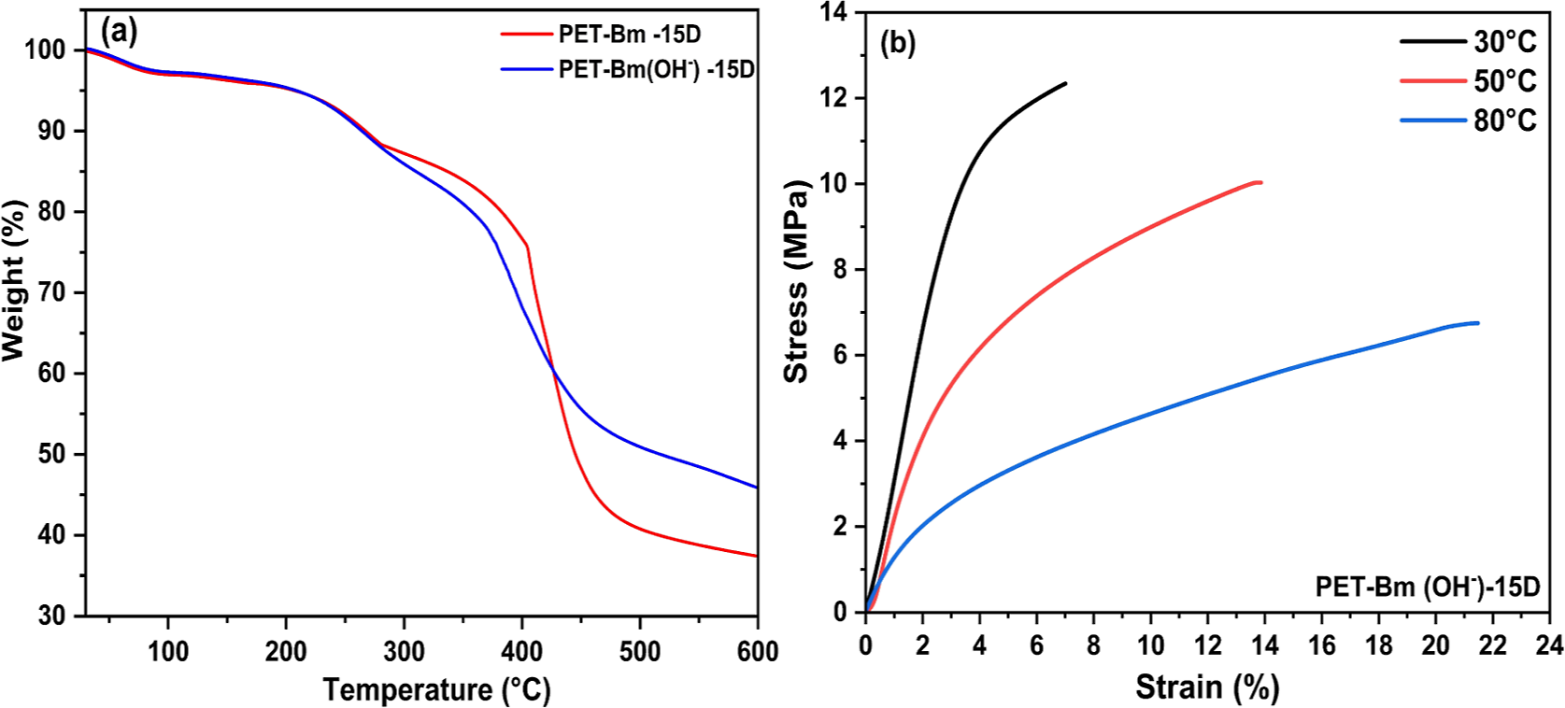
(a) TGA thermograms of PET-Bm and PET-Bm(OH^–^)-15D membranes. (b) Stress and strain curve for PET-Bm(OH^–^)-15D.

Figure 5a displays TGA thermographs of PET-Bm and PET-Bm(OH)-15D membranes, revealing three distinct degradation stages. Both membranes exhibit a small weight loss (3–4%) below 200 °C corresponding to the evaporation of moisture that is trapped inside the membranes during the washing step. A second weight loss of approximately 8% was observed within the temperature range of 205 to 250 °C suggesting the thermal degradation of the quaternary amine functional group.^59^ At 380 °C, a further weight loss of 45.82 and 33.89% was observed for PET-Bm and PET-Bm(OH)-15D, respectively, corresponding to the degradation of the polymer backbone.^60^ Considering that the working environment of AEM is usually at a temperature below 100 °C, it suggests that the membrane in OH form fully satisfies the thermal stability requirement of AEM.

The mechanical stability of the membrane in OH form (PET-Bm(OH^–^)-15D) was evaluated through multiple tensile tests conducted at various temperatures (30, 50, and 80 °C). The corresponding stress–strain curves are depicted in Figure 5b. As expected, an increase in temperature caused a progressive softening of the membrane, with a reduction in elastic modulus and yield stress and an increase in strain at failure.^61^ This tensile behavior is typical of polymer membranes. Specifically, at 30 and 50 °C, the membrane displayed a harder and more brittle nature, while at 80 °C, it demonstrated a softer and weaker behavior. The average elastic moduli calculated are 359, 304, and 235 MPa at 30, 50, and 80 °C, respectively. Moreover, the elongation of the sample increased with temperature from 6% up to 21% at 80 °C. These findings align with the expected characteristics observed in polymer membranes, emphasizing the necessity of accounting for temperature effects when evaluating membranes’ mechanical properties and stability.^62^

#### 3.4.3 Chemical Stability

**Figure 6:**
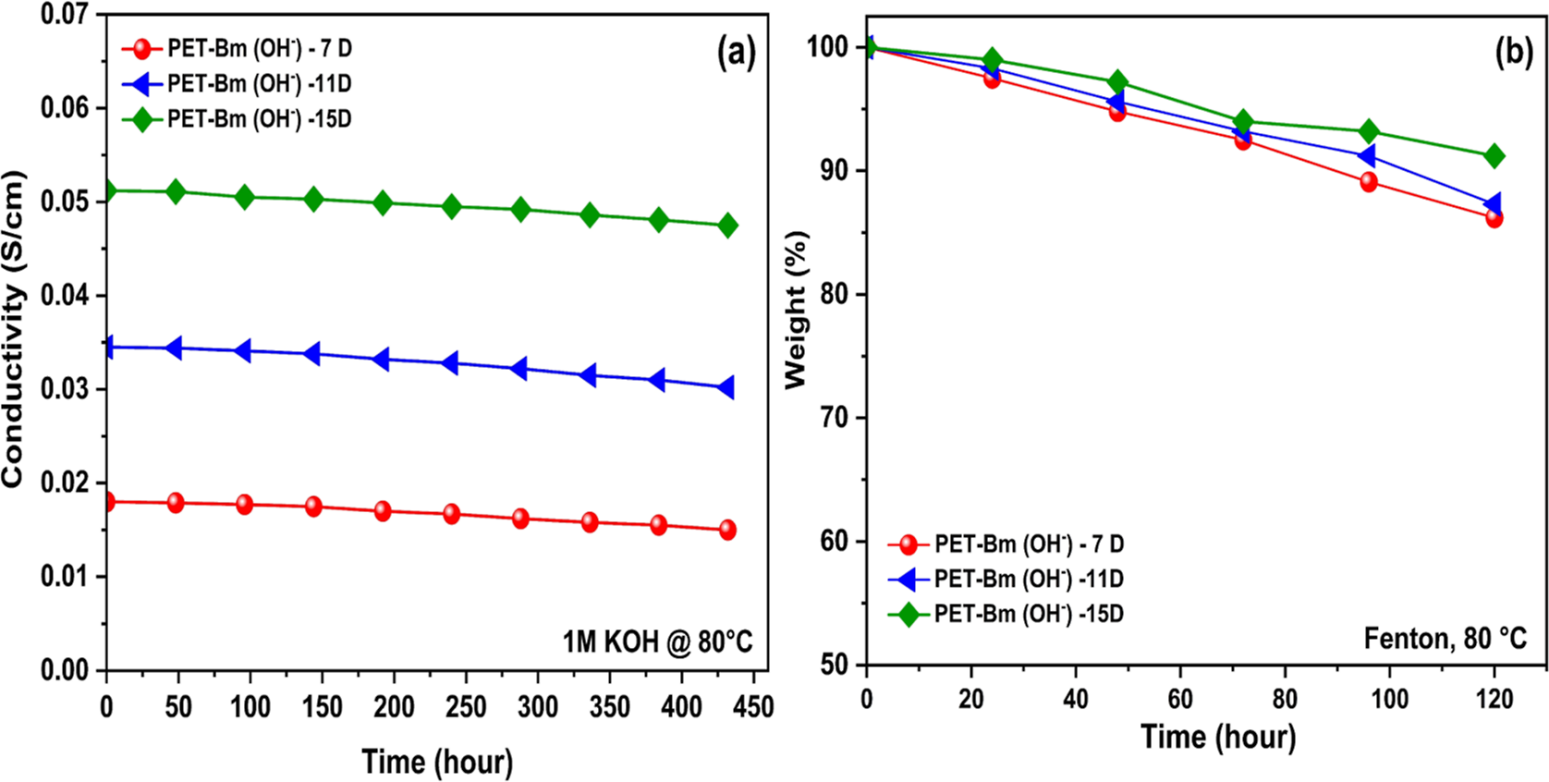
(a) Ionic conductivity of PET-Bm (OH^–^) (7 to 15D) at 80 °C with increasing immersion times in alkaline solution. (b) Oxidation stability of PET-Bm (OH^–^) (7 to 15D) in the Fenton reagent at 80 °C for 120 h.

The long-term AEMs stability is a crucial factor in the application of electrochemical devices, especially when they are required to operate for prolonged periods under elevated pH levels and temperatures.^63^ The stability of PET-Bm (OH^–^) at different degrees of amination (7, 11, and 15 days) was tested by immersing it in a strongly alkaline solution at 80 °C, and the conductivity was monitored regularly (Figure 6a). PET-Bm (OH^–^)-15D shows the highest alkaline stability of 432 h respected to other membranes (7D and 11D) due to the higher number of cationic groups in the modified PET structure. The amine functional groups present in PET-Bm(OH^–^)- are susceptible to nucleophilic substitution reactions in alkaline environments,^64^ forming new compounds and losing the original structure. This process results in a reduction of the cation head groups and subsequently affects the OH^–^ conductivity.^65^

In the development of durable AEMs, the choice of the base polymer backbone is critical as it influences the oxidative stability of the membrane against hydroxyl and peroxyl radical attacks.^66^ These radicals initiate the degradation process and can lead to the complete removal of functional groups. Hence, selecting a stable, functional group is equally important in improving the oxidative stability of AEMs. The oxidative stability of PET-Bm(OH^–^) membranes with varying degrees of amination time (7–15D) was assessed by subjecting them to Fenton’s reagent at 80 °C as shown in Figure 6b. A gradual decrease in weight loss of membranes over time was observed, particularly within the initial 42 h, indicating their susceptibility to radical attack. However, the finding also suggests that the PET-Bm(OH^–^) with longer amination time exhibits improved resistance to oxidative degradation as evidenced by an increase in remaining mass from 86.2 to 91.21% between 7 and 15 days.

**Figure 7:**
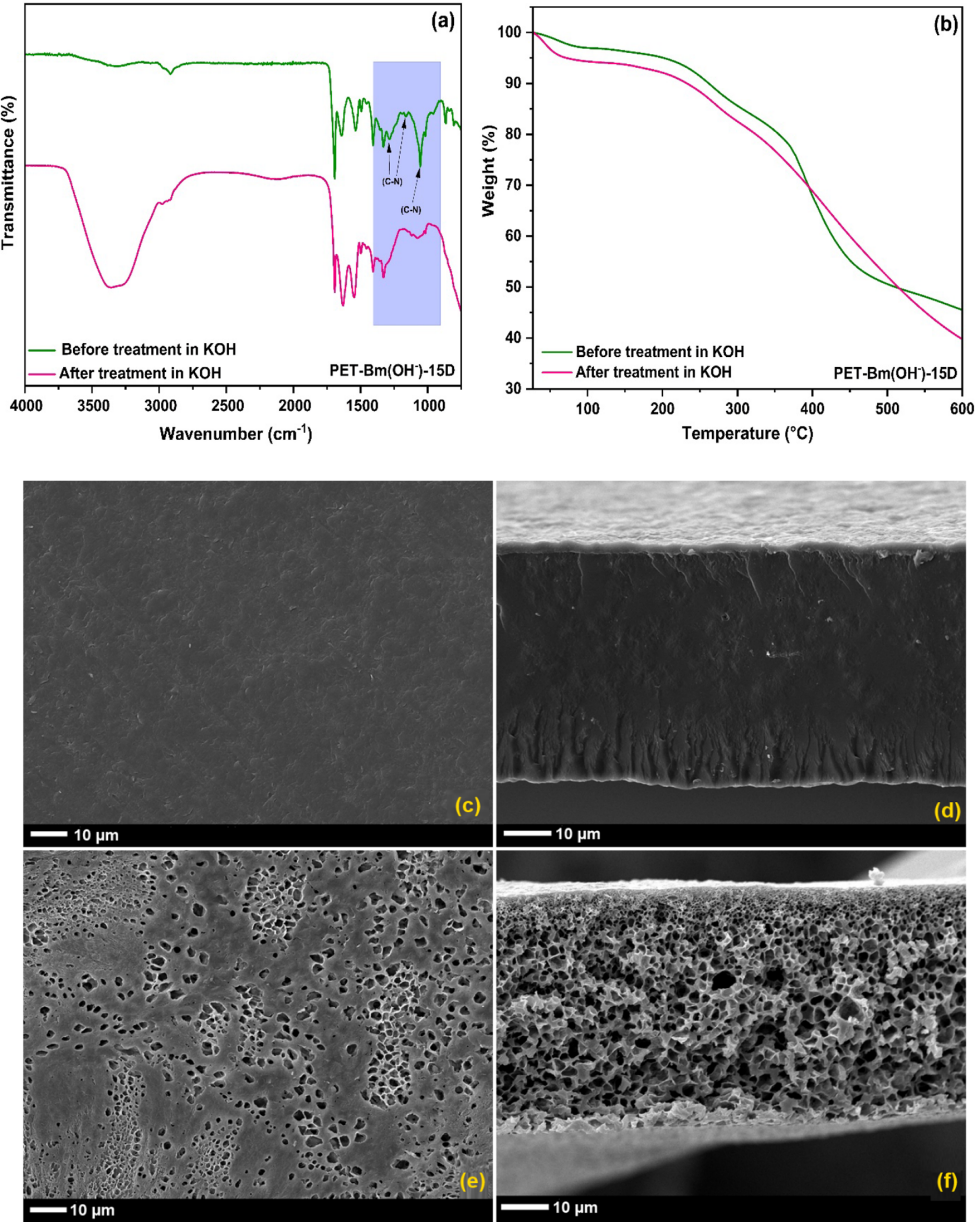
(a) FTIR spectra, (b) TGA thermograms of PET-Bm (OH^**–**^) and SEM micrographs (c,d) before and (e,f) after alkaline treatment (1 M KOH at 80 °C, 432 h).

FTIR analysis was conducted to verify the chemical structure changes in the membrane caused by the alkaline treatments, as depicted in Figure 7a. The absence of C–N stretching frequencies at 1060, 1165, and 1283 cm^–1^ indicates the loss of amine functional groups,^67^ which is the primary cause of the decline in conductivity. To further verify the stability of the membrane, TGA was carried out on the membrane after alkaline treatment to complement the FTIR. When the harsh alkaline condition was applied, the magnitude of the weight loss was increased (Figure 7b), which is correlated with the loss of quaternary ammonium cations. Furthermore, a reduction in the dimensional stability was observed for the degraded membrane. The SEM micrographs of the membrane before alkaline treatment show a compact and uniform morphology (Figure 7c). Additionally, the cross-section of the membrane displays no signs of cracks or pores (Figure 7d), indicating good compatibility among its components. However, upon alkaline degradation, the membrane exhibits a porous morphology resulting from the nucleophilic attacks, where OH^–^ ions serve as nucleophiles and react with electrophilic sites of AEM. This reaction leads to the formation of defects and porosity, which disrupt the membrane’s uniformity, as shown in Figure 7e,f. Consequently, these defects hinder the normal OH^–^ transport mechanism and result in a subsequent reduction in conductivity. Furthermore, irregular pores make the membrane prone to deformation and mechanical failure.

TSA was conducted to estimate and compare activation energies and reaction energies of potential chemical degradation mechanisms. OH^–^ attack causes the progressive degradation of the membranes through the loss of amine functional groups, as proved by FTIR measurement. However, the methylated membrane can undergo different degradation reactions under alkaline conditions. Nucleophilic S_N_2 substitution, Ylide formation, and Hoffman elimination are typical nucleophilic reactions in trimethylammonium (TMA) functionalized AEMs.^63,68^

**Scheme 3:**
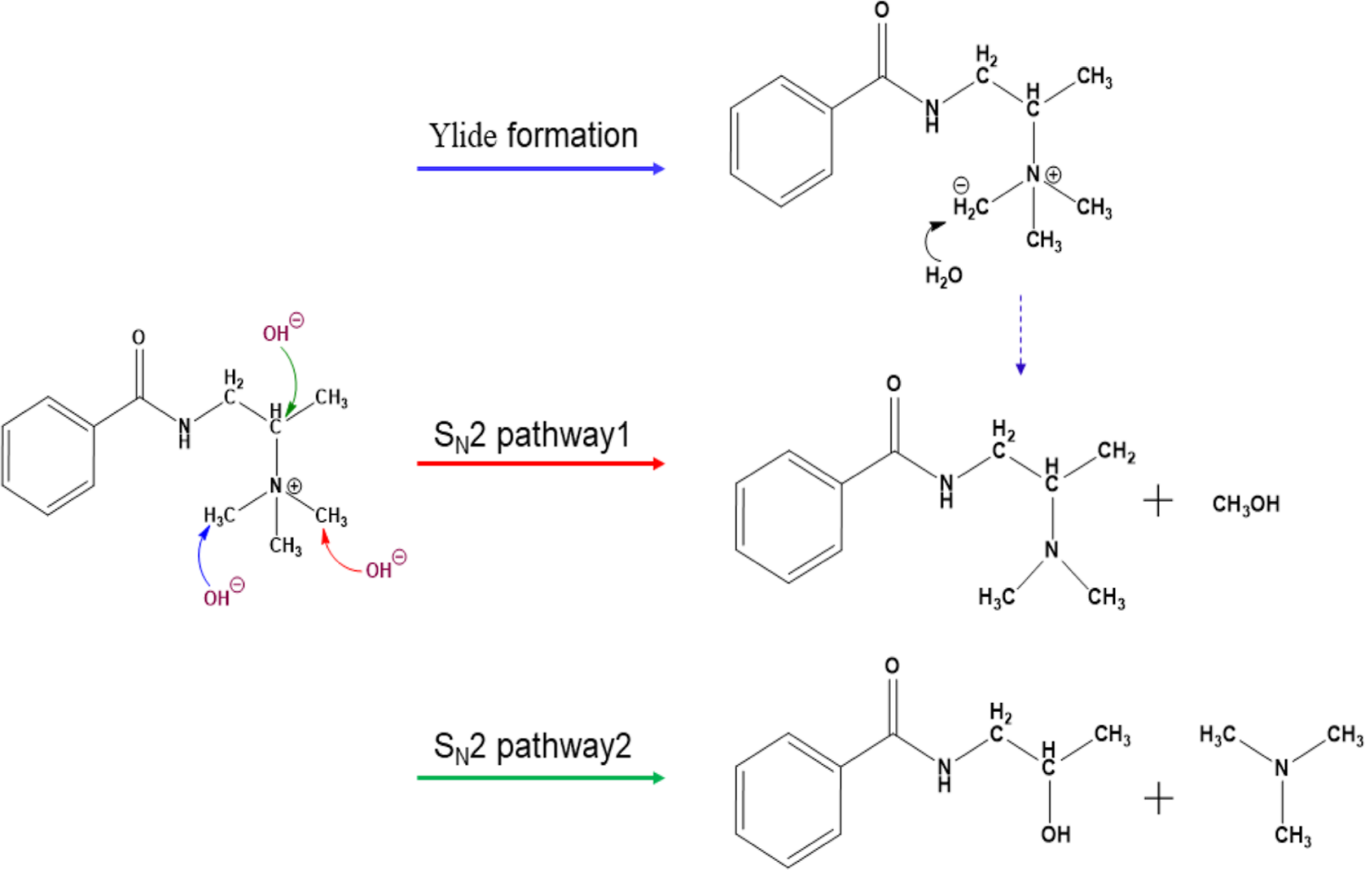
Proposed Alkaline Degradation Mechanisms: Ylide Formation, S_N_2 pathway1, and S_N_2 pathway2

A deeper understanding of the degradation mechanism can be provided by DFT analysis. PET-B’s initial and final structures interacting with OH^–^ were optimized for each simulated reaction at the B3LYP/6-311G level. A lower-level basis set was selected to reduce the computational costs. Due to structure symmetry, only half of the monomer structure was considered. Four H_2_O molecules were explicitly included around the hydroxide anion,^65,69^ forming a 4-coordinated OH^–^ reactant (OH^–^(H_2_O)_4_) to account for the screening effect of water on OH^–^ polymer interaction. To predict the transition states (TS), the climbing image Nudged Elastic Band method with TS optimization (NEB-TS) was implemented in ORCA. Vibrational frequencies were calculated to verify the presence of a single imaginary frequency value, proving that TS structures were actual saddle points in the minimum energy path (MEP). This paper proposes three degradation routes as shown in Scheme 3: two different pathways for S_N_2 reaction and the Ylide formation mechanism. Given the absence of β-H in the alkyl chain, the Hoffmann elimination reaction has not been considered.^70^ The S_N_2 pathway1 reaction involves the OH^–^ attack of an α-C in a methyl group of the TMA, with the release of methanol. Alternatively, the S_N_2 pathway2 occurs by means of an OH^–^ attack to the α-C attached to the backbone, with the final separation of the N(CH_3_)_3_ group. The Ylide formation mechanism, instead, involves the attack of an α-H in the TMA head group with the evolution of a water molecule. The ylide compound is very unstable and leads to further side reactions, such as the formation of methanol as in the S_N_2 pathway1.

**Figure 8:**
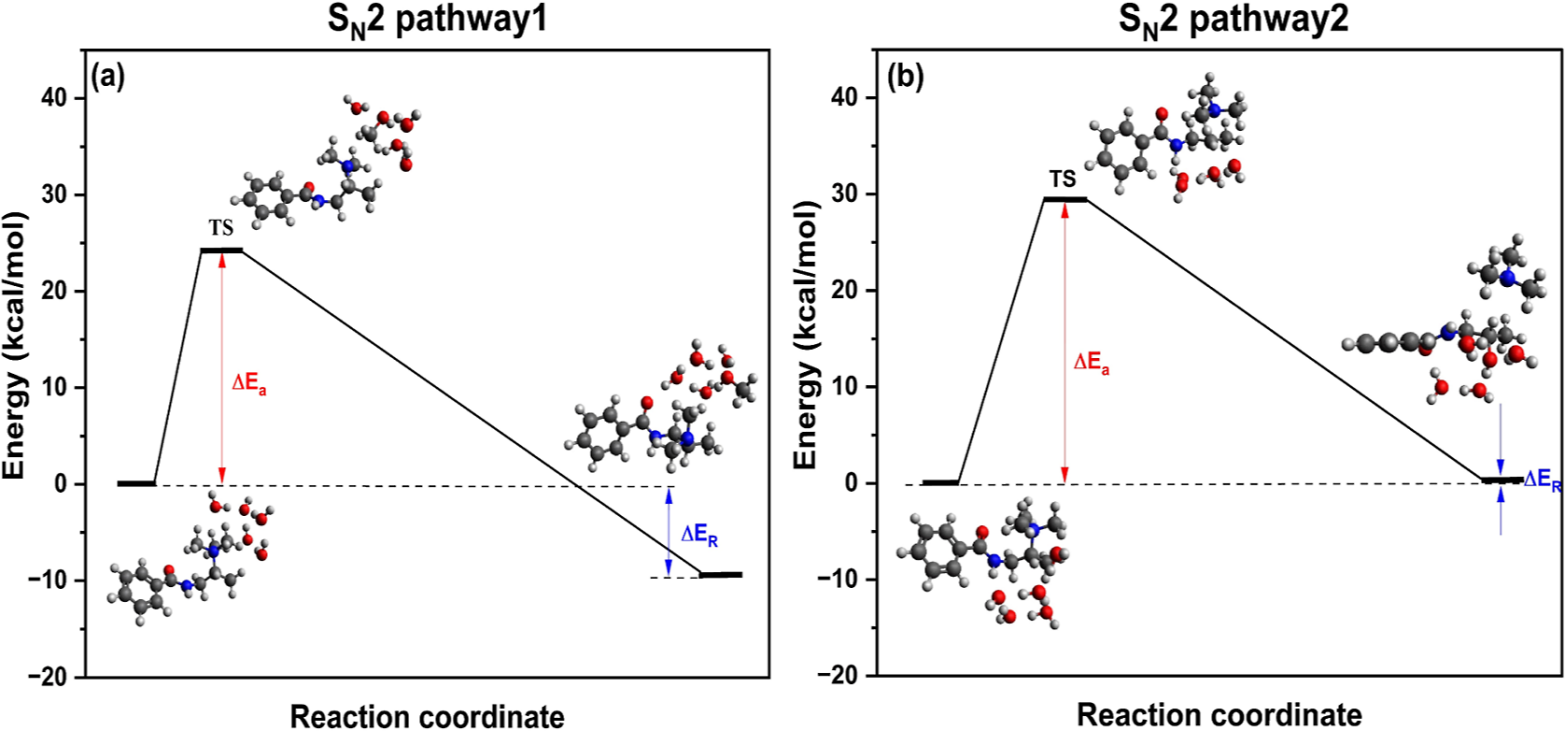
Schematic representation of minimum energy paths and geometries along (a) S_N_2 pathway1 and (b) S_N_2 pathway2 mechanisms in hydrated conditions.

The results of Figure 8a,b, as also shown in Table S4, demonstrate that a lower activation energy was required for S_N_2 pathway1 (Δ*E*_a_ = 24.23 kcal/mol) compared to S_N_2 pathway2 (Δ*E*_a_ = 29.39 kcal/mol), while Ylide formation was not observed in hydrated conditions. Given that this is a very unstable intermediate, we could not minimize a geometry corresponding to the ylide. Moreover, in the S_N_2 pathway2 reaction, Δ*E*_R_ is slightly endothermic (0.88 kcal/mol), indicating a destabilization of the water molecules in the product.^65^ Therefore, we conclude that the S_N_2 pathway1 mechanism is favored in hydrated conditions. This outcome confirmed the FTIR results (Figure 7a), which proved the loss of C–N bonds after chemical degradation.

## 4 Conclusions

This study demonstrates the feasibility of the anion exchange membrane preparation via two-step chemical modification of PET bottles. Initially, ethylene glycol was incorporated into PET then it was functionalized with 1,2-diaminopropane. The solution casting technique was applied to prepare AEM, wherein the QA group was incorporated into the modified PET-B through methylation and followed by a subsequent OH^–^ exchange process. FTIR and NMR analysis provides direct experimental evidence for modified PET’s functionalization and molecular structure. Additionally, ab-initio DFT analysis is a complementary tool to the experimental results, validating and supporting these findings. The synthesis process was optimized by improving the degree of amination, which was found to have a positive impact on IEC, WU, SR, and conductivity. The resulting values of 29.3 and 4.20% were achieved at 80 °C for WU and SR, respectively, whereas the highest value of IEC was 1.35 mmol·g^–1^ for the membrane with 80% amination. These results suggest that the amination process enhances the water channel structure, creating a preferential OH^–^ transport pathway. Conductivity is a crucial parameter for the performance of AEMs, and the highest conductivity was found to be 5.3 × 10^–2^ S·cm^–1^ at 80 °C. The conductivity of the membrane with higher IEC was consistent with the observed trend in water uptake. The membrane demonstrates 432 h of durability in 1 M KOH at 80 °C. Furthermore, longer amination time exhibits improved resistance to oxidative degradation. The loss of the QA group was verified with FTIR, TGA, and SEM analysis. Additionally, computational chemistry analysis was performed to investigate possible degradation mechanisms via a TSA. The results proved that the S_N_2 pathway, with the release of methanol molecules, is the preferred reaction in hydrated conditions. Although more investigations are required on the performance of this membrane in an assembled cell, the results obtained show the potential of recycled PET bottles as raw material for membranes in electrochemical applications such as AEM fuel cells, AEM water electrolyzers, or vanadium redox flow batteries, which could contribute to the development of a more sustainable and circular economy, while also saving the costs of development and production of the polymer. The chemical modification process was useful for producing OH^–^ conductive and effective membranes, whose properties could further increase through research and development for industrial implementation.

## Supporting Information Available

Reaction scheme mechanism of PET with ethylene glycol forming PET; formula and calculations for percentage of PET conversion and yield of PET-A; optimization reaction for PET-A and PET-B; elemental analyses for all the compounds; ^1^H NMR and ^13^C NMR for PET-A samples: FT-IR and NMR peak observation and simulated PET-B spectra for all the compounds; activation energy (Δ*E*_a_) and reaction energy (Δ*E*_R_) values for S_N_2 pathway1 and S_N_2 pathway2 (PDF)
