# Supplementary material for: Anion Exchange Membranes Based on Chemical Modification of Recycled PET Bottles
Source: ACS Appl Polym Mater. 2023 Aug 30;5(9):7548–61. doi: 10.1021/acsapm.3c01391 (PMC10496110; doi:10.1021/acsapm.3c01391)
Supplement: Supplementary file 1 — ap3c01391_si_001.pdf [file ap3c01391_si_001.pdf]

Supporting Information

Anion exchange membranes based on chemical modification of recycled PET bottles

Varun Donnakatte Neelalochana[a], Eleonora Tomasino[a], Rosa Di Maggio[a], Oscar Cotini[a], Paolo Scardi[a], Stefano Mammi[b], Narges Ataollahi[a]*

[*]*Corresponding Author:* *narges.ataollahi@unitn.it*

[a] *Department of Civil, Environmental and Mechanical Engineering, University of Trento, Via Mesiano 77, 38123, Trento, Italy*

[b] *Department of Chemical Sciences, University of Padova, Via Marzolo 1, 35131, Padova, Italy*

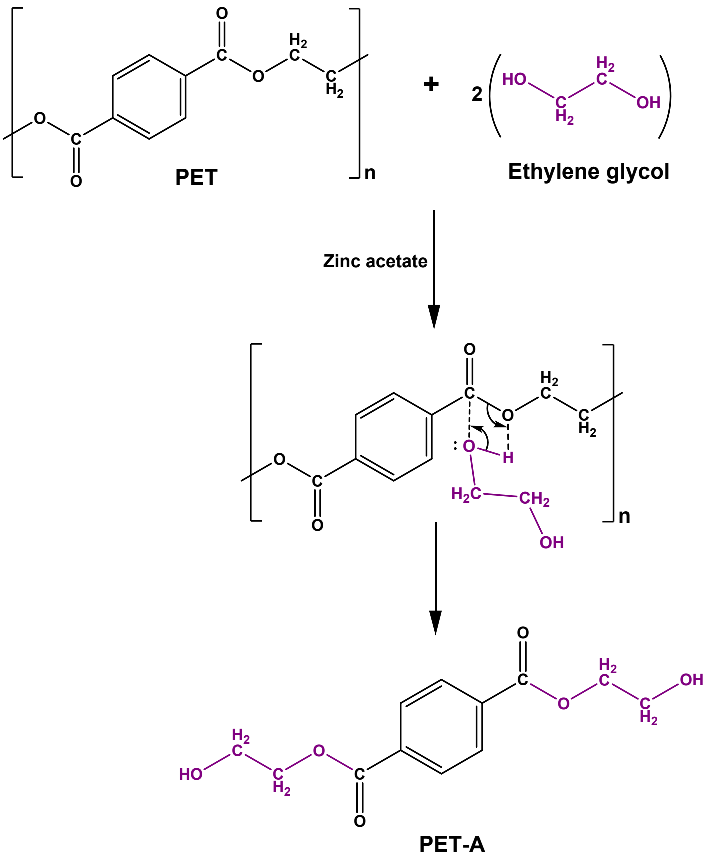

Scheme S1: The reaction mechanism of PET with ethylene glycol forming PET-A (Step-1).

The percentage of PET conversion and yield of PET-A were calculated according to formulas 1 and 2, respectively.

$$\text{PET Conversion (\%)} = \frac{W_0 - W_1}{W_0} \times 100\ \% \qquad (1)$$

$$\text{Yield of PET-A (\%)} = \frac{W_{PET.A} \times MW_{PET.A}}{W_{PET.I} \times MW_{PET}} \times 100\ \% \qquad (2)$$

Where W_0 and W_1 represent the initial weight of PET and un-depolymerized PET, respectively. The terms W_{PET} and $W_{PET.A}$ refers to the initial weight of PET and the obtained weight of PET-A, respectively. MW_{PET} and $MW_{PET.A}$ denote the molecular weight of the repeating units of PET (192 g/mol) and PET-A (254 g/mol), respectively.

The effect of reaction temperature and time on the parameters of PET conversion and yield, which determine the efficiency of a chemical reaction, was investigated, and presented in Figure S1 and Table S1. The percentage of transformed reactant is conversion, while yield is the percentage of the obtained product with respect to the potential amount. Figure S1a indicates that increasing the reaction temperature from 160 °C to 190 °C significantly increased PET conversion and yield. The optimal conversion rate (94.5%) and yield (76.2%) were attained at 180 °C. It was observed that the reaction time is also critical, and the conversion of PET-A increases with it. The conversion reached 93.2% with the highest yield (75.8%) after 4 hours of reaction. However, the yield of PET-A decreased as the reaction time exceeded 5 hours, which can be attributed to the formation of side reactions.

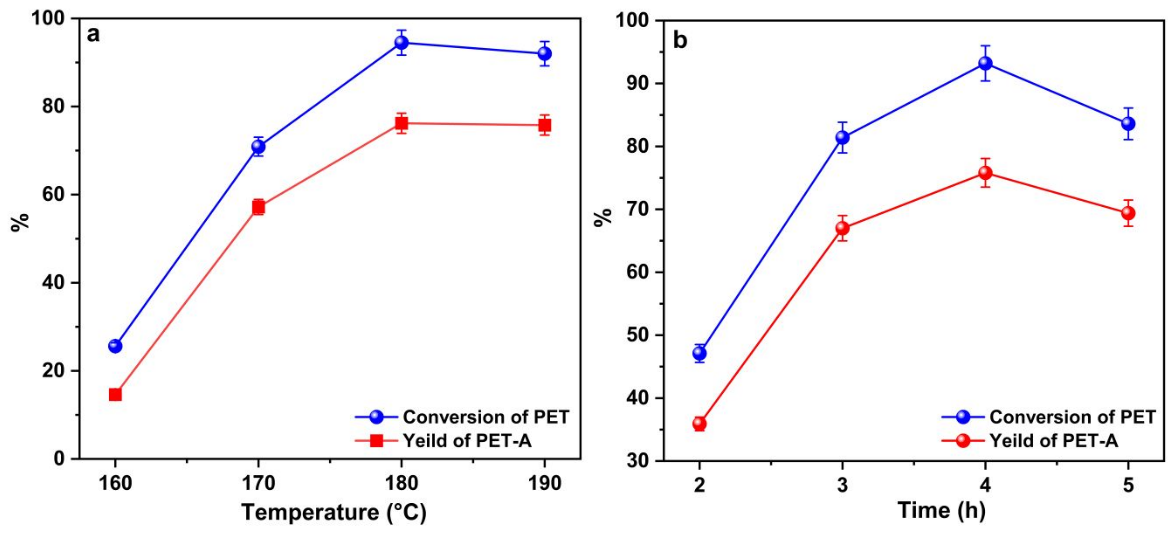

Figure S1: Optimization of reaction: (a) Effect of temperature (160 °C-190 °C, 4 h) on PET-A, (b) Effect of reaction time (1–5 h, 180 °C) on PET-A. (Reaction conditions: PET: EG=1:3, zinc acetate (0.2 wt.%).

Table S1: Composition of the samples (C and H) based on Elemental analyses. (%O was obtained as a difference of 100)

Samples	C (±0.1%)	H (±0.1%)	O# (±0.1%)	N (±0.1%)	C/O	C/N	Yield (±0.2%)	Conversion (±0.2%)
**Theoretical PET**	57.09	4.03	38.88	/	1.46	/	/	/
**PET bottle**	61.42	5.03	33.55	/	1.83	/	/	/
**PET-A (2 hours)**	57.95	6.09	35.96	/	1.61	/	35.90	47.10
**PET-A (3 hours)**	56.06	6.62	37.32	/	1.50	/	67.01	81.42
**PET-A (4 hours)**	55.99	6.61	37.40	/	1.49	/	75.80	93.21
**PET-A (5 hours)**	53.91	7.02	39.07	/	1.37	/	69.40	83.60
**PET-B (7 days)**	54.57	8.11	19.96	17.36	2.73	3.14	54.18	50.10
**PET-B (11 days)**	58.34	9.24	14.05	18.37	4.15	3.17	62.18	66.36
**PET-B (15 days)**	59.89	9.06	11.62	19.43	5.15	3.08	69.92	80.05

#**%O** was obtained as a difference of 100.

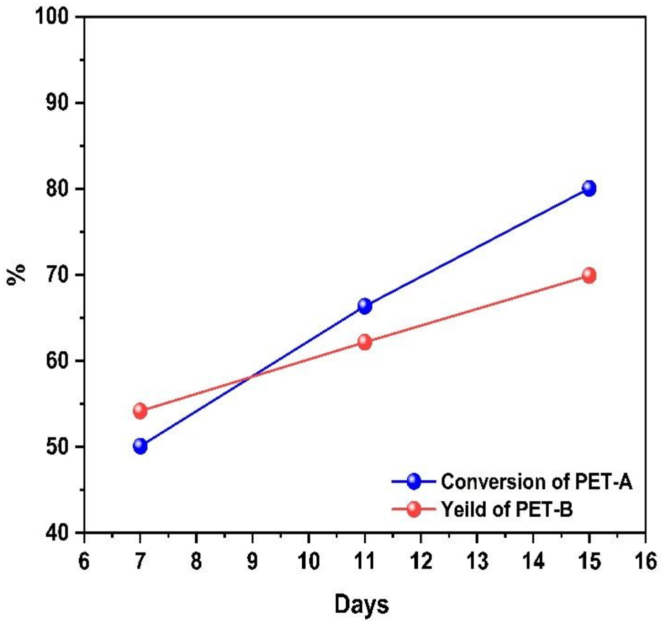

Figure S2: Optimization of reaction: Effect of reaction time (7-15 days) on PET-B.

NMR analysis

The ^{1}H-NMR spectrum of PET-A showed a signal at 1.99 ppm corresponding to hydroxyl proton. The singlet at 3.98 and triplet at 4.49 ppm belong to the methylene protons CH_2OH and $COO\text{-}CH_2$, respectively. A singlet peak of the aromatic ring was observed at 8.12 ppm. The signals observed at 1.58 ppm and 7.27 ppm were assigned to the residual water and $CDCl_3$ solvent, respectively. The ^{13}C-NMR spectrum of PET-A, shown in Figure S3, supports the ^{1}H-NMR analysis. The observed signals at 60.2 ppm, 66.5 ppm, and 129.8 ppm correspond to carbon atoms of the aliphatic CH_2 groups and the protonated aromatic carbons, respectively. The carbonyl carbon can generally be observed at a higher field (165-200 ppm).[1] These findings are in agreement with the literature.[2,3]

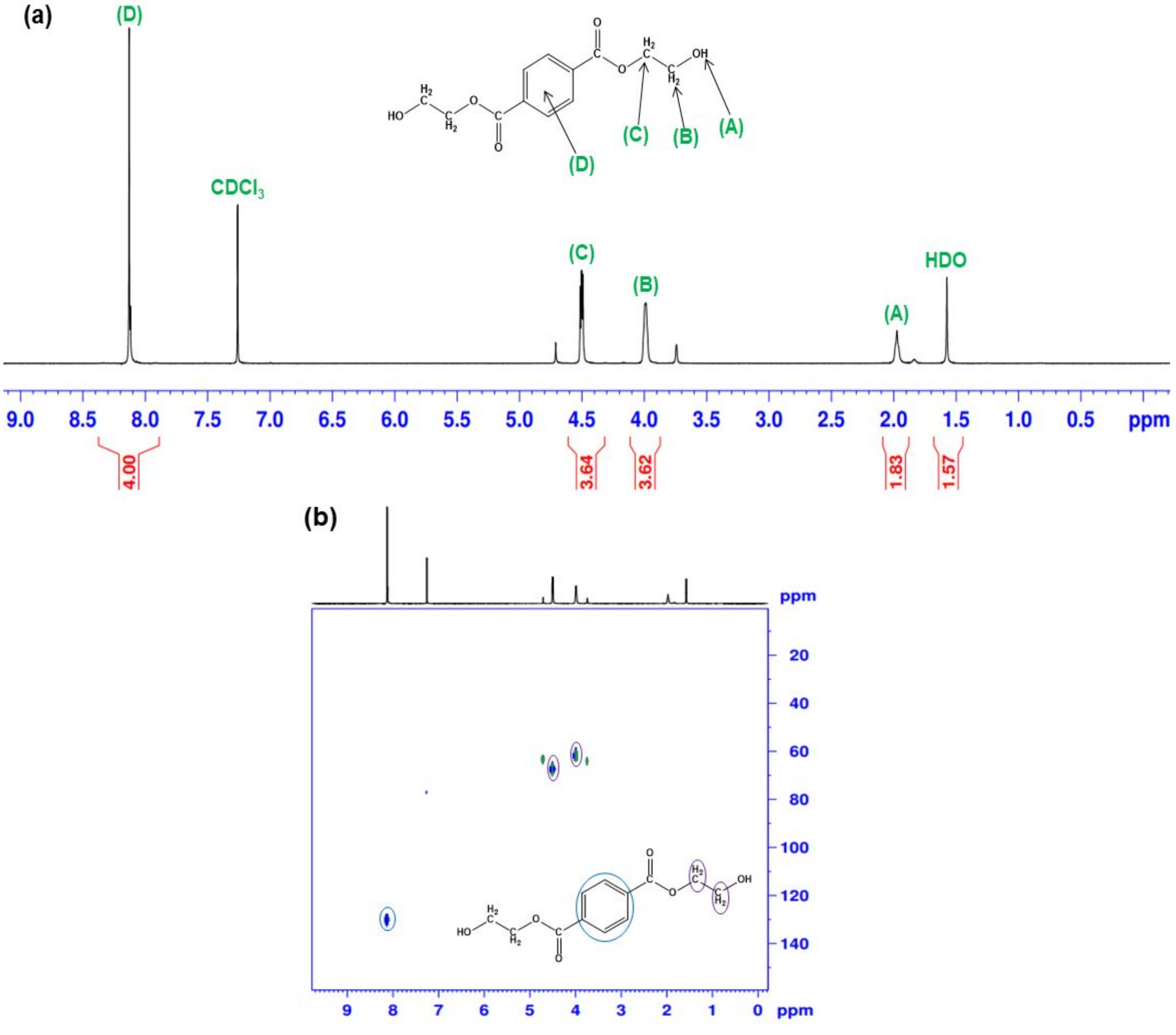

Figure S3: NMR spectra of PET-A: (a) ^{1}H-NMR and (b) ^{13}C NMR.

Table S2: FT-IR peak observation of PET, PET-A, PET-B, PET-Bm(OH$^-$) and

Wavenumber (cm^{-1}) & intensity					Band assignments	Reference
PET	PET-A	PET-B	PET-Bm (OH$^-$)	Simulated PET-B		
		633 (sh,m)			*v* (CH_2)	4,5
		692 (s)		672 (s)	*v* (C-N)	4,6
723 (s)	722 (vs)	722 (s)	728 (s)	747 (s)	δ (C-H) oop of ring	7–9
793 (vw)	792 (vw)	794 (m)			δ (C-H) oop of ring	7–9
		819 (m)	807 (m)	819 (m)	*v* (C-N)	4,5
		830 (sh,w)			*v* (C-H)	1,9
	836 (w)	838 (w)	836 (w)		*v* (C-C) of ring	10,11
846 (w)					*r* (CH_2)	4,12
	861 (m)	864 (m)	866 (m)	895 (m)	*v* (C-C) of ring	13,14
872 (w)	872 (m)				*r* (CH_2)	12,13
	897 (w)				*v* (C-O)	15
		931 (vw)			δ (C-H)	4,9
973 (vw)	975 (w)	978 (m)	965 (m)	980 (w)	*v* (C=C) of ring	10,12,14
1020 (w)	1019 (m)				*v* (O-C-C)	10,16
1044(sh,vw)					*v* (C-O)	12,13
	1067 (s)				*v* (C-O)	13,17
1093 (w)					*v* (C-O)	12,18
1115 (sh,w)	1111 (m)				*v* (C-O)	12,18,19
	1128 (m)				*v* (C-O)	13,17
		1165 (w)	1163 (w)	1074 (m)	*v* (C-N)	4,6
1240 (w)	1248 (s)				*v* (C-O-C)	12,18,19
	1271 (s)				*v* (C-O)	19
		1283 (m)	1282 (m)	1217 (s)	*v* (C-N)	6,20
		1292 (m)			*v* (C-N)	4,5,20
		1313 (m)	1320 (m)		*v* (C-N)	4,5
		1335 (m)			*v* (C-N)	4,5,20
1340 (w)	1343 (w)				*v* (C-C) of ring	13,14
	1357 (vw)	1359 (m)	1355 (w)	1305 (w)	δ (CH_2)	4,5
1373 (vw)	1375 (w)	1373 (m)		1337 (w)	*w* (CH_2)	1,12
		1397 (w)			*v* (C-H)	1,21
1408 (w)	1408 (m)				*v* (C-C) of ring	8,12

simulated PET-B spectra obtained with DFT analysis.

		1438 (w)	1426 (w)		δ (CH_2)	9
1456 (vw)	1456 (w)	1456 (m)	1454 (w)	1412 (sh)	δ (CH_2)	8,12,14
1503 (vw)	1503 (vw)	1500 (m)	1501 (m)	1427 (sh)	*v* (C=C)	43,45
		1539 (s)	1537 (s)	1445 (vs)	*v* (N-H)	10,22,23
1588 (vw)	1588 (vw)	1588 (m)		1547 (m)	*v* (C=C)	10,12
		1632 (s)	1636 (vs)	1615 (vs)	*v* (C=O)	21,24
	1687 (s)		1692 (vs)		*v* (C=C)	21,24
1714 (m)	1714 (vs)				*v* (C=O)	8,21,25
	2881 (m)	2879 (s)	2879 (w)	2710 (s)	*v* (CH_2)	4,9
2907 (vw)					*v* (C-H)	4
	2930 (m)	2930 (s)	2925 (m)	2841 (s)	*v* (C-H)	13,22
	2947 (m)				*v* (C-H)	19,26
2971 (w)	2971 (m)	2971 (vs)		2909 (m)	*v* (C-H)	14,22
		3030 (m)	3039 (w)	2997 (m)	*v* (C-H)	4,5
	3072 (sh,w)	3072 (m)			*v* (C-H)	4,5
		3283 (vs)	3321 (w)	3407 (w)	δ (N-H)	10,22,23
	3439 (vs)		3432 (w)		*v* (O-H)	7,19,27

Relative intensities are reported in parentheses: vs: very strong; s: strong; m: medium; w: weak; vw: very weak; sh: shoulder. *v*: stretching; δ: bending; *w*: wagging; oop: out-of- plane; *r*-rocking.

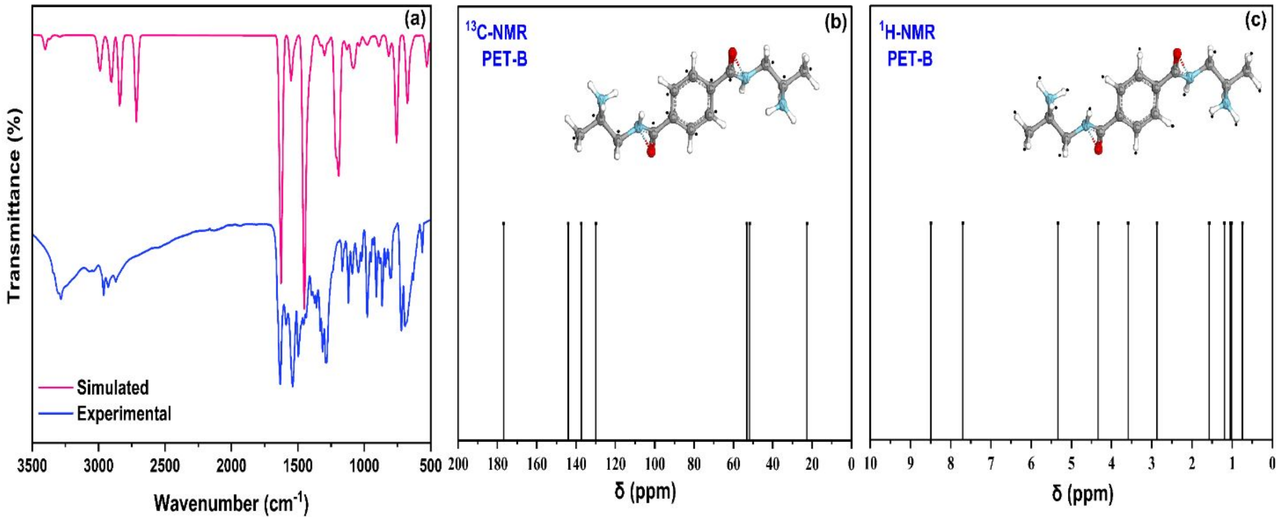

Figure S4: (a) Comparison of computed (pink) and experimental (blue) FTIR spectra of PET-B. (b) Simulated chemical shifts of ^{13}C-NMR and (c) ^{1}H-NMR.

Table S3: NMR peak observation of Simulation and Experimental for PET-B.

^{1}H-NMR δ (ppm)		
**Simulation**	**Experimental**	**Peak assignment**
0.74	0.96	methyl CH_3
1.02 – 1.21	1.10-1.20	methyl CH_3
1.55	1.80	NH_2
-	2.49	DMSO
2.88	2.95	CH-CH_3
3.59	3.15-3.49	CH_2-CH_3
4.34	-	CH
5.32	-	CH
7.70	7.89	aromatic ring
8.49	8.50	NH

^{13}C-NMR δ (ppm)		
**Simulation**	**Experimental**	**Peak assignment**
22.5	20.8	CH_3
51.5	40.1	CH
53.0	49.5	CH_2
129.8-143.8	127.6	aromatic ring
177.01	-	C=O

Table S4: Calculated activation energy (ΔEa) and reaction energy (ΔER) values for S_N2 pathway1 and S_N2 pathway2.

Mechanism	ΔE_a [kcal/mol]	ΔE_R [kcal/mol]
**S_N2 pathway1**	24.23	-9.53
**S_N2 pathway2**	29.39	0.88

Reference

(1) Bruno, T. J.; Svoronos, P. D. N. *CRC Handbook of Basic Tables for Chemical Analysis*; 2003. https://doi.org/10.1201/9781420039917.

(2) Ghaemy, M.; Mossaddegh, K. Depolymerisation of Poly(Ethylene Terephthalate) Fibre Wastes Using Ethylene Glycol. *Polym. Degrad. Stab.* **2005**, *90* (3), 570–576. https://doi.org/10.1016/j.polymdegradstab.2005.03.011.

(3) Lima, G. R.; Monteiro, W. F.; Scheid, C. M.; Ligabue, R. A.; Santana, R. M. C. Evaluation of Sodium/Protonated Titanate Nanotubes Catalysts in Virgin and Post Consumer PET Depolymerization. *Catal. Letters* **2019**, *149* (5), 1415–1426. https://doi.org/10.1007/s10562-019-02724-8.

(4) Workman, J. 'The Handbook of Organica Compounds: NIR, IR, Raman, and UV-Vis Spectra Feautiring Polymers and Surfactants'. **2001**.

(5) Larkin, P. J. *Infrared and Raman Spectroscopy: Principles and Spectral Interpretation*; 2017. https://doi.org/10.1016/C2015-0-00806-1.

(6) Ataollahi, N.; Vezzù, K.; Nawn, G.; Pace, G.; Cavinato, G.; Girardi, F.; Scardi, P.; Di Noto, V.; Di Maggio, R. A Polyketone-Based Anion Exchange Membrane for Electrochemical Applications: Synthesis and Characterization. *Electrochim. Acta* **2017**, *226*, 148–157. https://doi.org/10.1016/j.electacta.2016.12.150.

(7) Fadel, M. A.; Kamel, N. A.; Darwish, M. M.; El-Messieh, S. L. A.; Abd-EL-Nour, K. N.; Khalil, W. A. Preparation and Characterization of Polyethylene Terephthalate–Chamomile Oil Blends with Enhanced Hydrophilicity and Anticoagulant Properties. *Prog. Biomater.* **2020**, *9* (3), 97–106. https://doi.org/10.1007/s40204-020-00133-4.

(8) El-Saftawy, A. A.; Elfalaky, A.; Ragheb, M. S.; Zakhary, S. G. Electron Beam Induced Surface Modifications of PET Film. *Radiat. Phys. Chem.* **2014**, *102*, 96–102. https://doi.org/10.1016/j.radphyschem.2014.04.025.

(9) Md Salim, R.; Asik, J.; Sarjadi, M. S. Chemical Functional Groups of Extractives, Cellulose and Lignin Extracted from Native Leucaena Leucocephala Bark. *Wood Sci. Technol.* **2021**, *55* (2), 295–313. https://doi.org/10.1007/s00226-020-01258-2.

(10) Mersha, D. A.; Sendekie, Z. B. High-Temperature Performance Enhancement of Bitumen by Waste PET-Derived Polyurethane. *Adv. Mater. Sci. Eng.* **2022**, *2022*. https://doi.org/10.1155/2022/9567197.

(11) Zhou, X.; Lu, X.; Wang, Q.; Zhu, M.; Li, Z. Effective Catalysis of Poly(Ethylene Terephthalate) (PET) Degradation by Metallic Acetate Ionic Liquids. *Pure Appl. Chem.* **2012**, *84* (3), 789–801. https://doi.org/10.1351/PAC-CON-11-06-10.

(12) Donelli, I.; Freddi, G.; Nierstrasz, V. A.; Taddei, P. Surface Structure and Properties of

Poly-(Ethylene Terephthalate) Hydrolyzed by Alkali and Cutinase. *Polym. Degrad. Stab.* **2010**, *95* (9), 1542–1550. https://doi.org/10.1016/j.polymdegradstab.2010.06.011.

(13) Chirea, M.; Freitas, A.; Vasile, B. S.; Ghitulica, C.; Pereira, C. M.; Silva, F. Gold Nanowire Networks: Synthesis, Characterization, and Catalytic Activity. *Langmuir* **2011**, *27* (7), 3906–3913. https://doi.org/10.1021/la104092b.

(14) More, A. P.; Kute, R. A.; Mhaske, S. T. Chemical Conversion of PET Waste Using Ethanolamine to Bis(2-Hydroxyethyl) Terephthalamide (BHETA) through Aminolysis and a Novel Plasticizer for PVC. *Iran. Polym. J. (English Ed.* **2014**, *23* (1), 59–67. https://doi.org/10.1007/s13726-013-0200-0.

(15) Dos Santos Pereira, A. P.; Da Silva, M. H. P.; Lima, É. P.; Dos Santos Paula, A.; Tommasini, F. J. Processing and Characterization of PET Composites Reinforced with Geopolymer Concrete Waste. *Mater. Res.* **2017**, *20*, 411–420. https://doi.org/10.1590/1980-5373-MR-2017-0734.

(16) Kárpáti, L.; Fogarassy, F.; Kovácsik, D.; Vargha, V. One-Pot Depolymerization and Polycondensation of PET Based Random Oligo- and Polyesters. *J. Polym. Environ.* **2019**, *27* (10), 2167–2181. https://doi.org/10.1007/s10924-019-01490-3.

(17) Mendiburu-Valor, E.; Mondragon, G.; González, N.; Kortaberria, G.; Eceiza, A.; Peña-Rodriguez, C. Improving the Efficiency for the Production of Bis-(2-Hydroxyethyl) Terephtalate (BHET) from the Glycolysis Reaction of Poly(Ethylene Terephtalate) (PET) in a Pressure Reactor. *Polymers (Basel).* **2021**, *13* (9). https://doi.org/10.3390/polym13091461.

(18) Yasir, A. H.; Khalaf, A. S.; Khalaf, M. N. Preparation and Characterization of Oligomer from Recycled PET and Evaluated as a Corrosion Inhibitor for C-Steel Material in 0.1 M HCl. *Open J. Org. Polym. Mater.* **2017**, *07* (01), 1–15. https://doi.org/10.4236/ojopm.2017.71001.

(19) Chinchillas-Chinchillas, M. J.; Orozco-Carmona, V. M.; Alvarado-Beltrán, C. G.; Almaral-Sánchez, J. L.; Sepulveda-Guzman, S.; Jasso-Ramos, L. E.; Castro-Beltrán, A. Synthesis of Recycled Poly(Ethylene Terephthalate)/Polyacrylonitrile/Styrene Composite Nanofibers by Electrospinning and Their Mechanical Properties Evaluation. *J. Polym. Environ.* **2019**, *27* (3), 659–669. https://doi.org/10.1007/s10924-019-01379-1.

(20) Dutt, K.; Soni, R. K. Synthesis and Characterization of Bis-Amino Ethyl Terephthalamide from PET Waste and Its Applications as Hardener in DGEBA. *Int. J. Plast. Technol.* **2014**, *18* (1), 16–26. https://doi.org/10.1007/s12588-014-9071-2.

(21) Li, R.; Leng, Z.; Yang, J.; Lu, G.; Huang, M.; Lan, J.; Zhang, H.; Bai, Y.; Dong, Z. Innovative Application of Waste Polyethylene Terephthalate (PET) Derived Additive as an Antistripping Agent for Asphalt Mixture: Experimental Investigation and Molecular Dynamics Simulation. *Fuel* **2021**, *300* (May), 121015. https://doi.org/10.1016/j.fuel.2021.121015.

(22) Kárpáti, L.; Fejér, M.; Kalocsai, D.; Molnár, J.; Vargha, V. Synthesis and Characterization of Isophorondiamine Based Epoxy Hardeners from Aminolysis of PET. *Express Polym. Lett.* **2019**, *13* (7), 618–631. https://doi.org/10.3144/expresspolymlett.2019.52.

(23) Kausar, A. Fabrication and Characteristics of Poly(Benzimidazole/Fluoro/Ether/Siloxane/Amide)/Sulfonated Polystyrene/Silica Nanoparticle-Based Proton Exchange Membranes Doped with Phosphoric Acid. *Int. J. Polym. Mater. Polym. Biomater.* **2015**, *64* (4), 184–191. https://doi.org/10.1080/00914037.2014.936589.

(24) Kaus, N. H.; Lahazan, N.; Ahmad, A. H. Ionic Conductivity in Poly (L-Leucine)1,3-Diamino Propane-Lithium Iodide Solid Polymer Electrolyte. *Polym. Adv. Technol.* **2009**, *20* (3), 156–160. https://doi.org/10.1002/pat.1246.

(25) Leng, Z.; Padhan, R. K.; Sreeram, A. Production of a Sustainable Paving Material through Chemical Recycling of Waste PET into Crumb Rubber Modified Asphalt. *J. Clean. Prod.* **2018**, *180*, 682–688. https://doi.org/10.1016/j.jclepro.2018.01.171.

(26) Aguado, A.; Becerra, L.; Martínez, L. Glycolysis Optimization of Different Complex PET Waste with Recovery and Reuse of Ethylene Glycol. *Chem. Pap.* **2022**, No. 0123456789, 1–21. https://doi.org/10.1007/s11696-023-02704-8.

(27) Jamdar, V.; Kathalewar, M.; Dubey, K. A.; Sabnis, A. Recycling of PET Wastes Using Electron Beam Radiations and Preparation of Polyurethane Coatings Using Recycled Material. *Prog. Org. Coatings* **2017**, *107*, 54–63. https://doi.org/10.1016/j.porgcoat.2017.02.007.
